# Meta-analysis of the association between variants in *MAPT* and neurodegenerative diseases

**DOI:** 10.18632/oncotarget.16690

**Published:** 2017-03-29

**Authors:** Cheng-Cheng Zhang, Jun-Xia Zhu, Yu Wan, Lin Tan, Hui-Fu Wang, Jin-Tai Yu, Lan Tan

**Affiliations:** ^1^ Department of Neurology, Qingdao Municipal Hospital, Dalian Medical University, Dalian, Liaoning, PR China; ^2^ Clinical Skills Training Center, Qingdao Municipal Hospital, Qingdao University, Qingdao, Shandong, PR China; ^3^ Department of Neurology, Qingdao Municipal Hospital, Qingdao University, Qingdao, Shandong, PR China; ^4^ College of Medicine and Pharmaceutics, Ocean University of China, Qingdao, Shandong, China

**Keywords:** *MAPT*, polymorphism, haplotype, neurodegenerative disease, meta-analysis

## Abstract

Microtubule-associated protein tau (MAPT) gene is compelling among the susceptibility genes of neurodegenerative diseases which include Alzheimer’s disease (AD), Parkinson’s disease (PD), progressive supranuclear palsy (PSP), corticobasal degeneration (CBD), frontotemporal dementia (FTD) and amyotrophic lateral sclerosis (ALS). Our meta-analysis aimed to find the association between MAPT and the risk of these diseases. Published literatures were retrieved from MEDLINE and other databases, and 82 case-control studies were recruited. Six haplotype tagging single-nucleotide polymorphisms (rs1467967, rs242557, rs3785883, rs2471738, del-In9 and rs7521) and haplotypes (H2 and H1c) were significantly associated with the above diseases. The odds ratios (ORs) and 95 % confidence intervals (CIs) were evaluated by comparison in minor and major allele frequency using the R software. This study demonstrated that different variants in MAPT were associated with AD (rs2471738: OR= 1.04, 95%CI = 1.00 - 1.09; H2: OR = 0.94, 95% CI = 0.91 - 0.97), PD (H2: OR = 0.76, 95% CI = 0.74 - 0.79), PSP (rs242557: OR = 1. 96, 95% CI = 1. 71 - 2.25; rs2471738: OR = 1. 85, 95% CI = 1. 48 - 2.31; H2: OR = 0.20, 95% CI = 0.18 - 0.23), CBD (rs242557: OR = 2.51, 95%CI = 1. 66 -3.78; rs2471738: OR = 2.07, 95%CI = 1. 32 -3.23; H2: OR = OR = 0.30, 95% CI = 0.23 - 0.41) and ALS (H2: OR = 0.92, 95% CI = 0.86 - 0.98) instead of FTD (H2: OR = 1.02, 95% CI = 0.78 - 1.32). In conclusion, MAPT is associated with risk of neurodegenerative diseases, suggesting crucial roles of tau in neurodegenerative processes.

## INTRODUCTION

Neurodegenerative diseases are a group of disorders with progressive neuronal loss in particular regions of brain, including Alzheimer's disease (AD), Parkinson's disease (PD), progressive supranuclear palsy (PSP), corticobasal degeneration (CBD), frontotemporal dementia (FTD), amyotrophic lateral sclerosis (ALS) and many others. The etiology of neurodegenerative diseases is complicated and multifactorial, mainly including genetic variants and environmental exposure. However, epidemiologic evidence for the association between the environmental exposure and neurodegenerative diseases is not conclusive [1]. Genetic variant is a crucial factor in etiology and pathogenic mechanisms of neurodegenerative diseases [2]. Hundreds of genetic variants have been confirmed significantly associated with neurodegenerative diseases, but the majority of these genes do not overlap across diseases [2]. Only several susceptibility genes relate to diverse neurodegenerative diseases. Among them, microtubule-associated protein tau (*MAPT*) gene is compelling [3]. Mutations in *MAPT* have been reported to participate in AD [4–6], PD [7–9], PSP [10, 11], CBD [12, 13], FTD [14, 15] and ALS [16, 17].

*MAPT* is located on chromosome 17q21.3, and encodes six tau isoforms ranging from 352 to 441 amino acids in length expressed in neurons [18]. To date, nearly 60 mutations in *MAPT* have been proved pathogenic to neurodegenerative diseases [3]. Most *MAPT* variants probably cause abnormal structure and function of hyperphosphorylated, insoluble and aggregated tau. These pathological changes are crucial for the pathogenesis of tau-related neurodegenerative diseases, which are called tauopathies [19]. As known to all, there are two main haplotypes in *MAPT*, H1 and H2. Several single-nucleotide polymorphisms (SNPs) throughout *MAPT* gene are in complete linkage disequilibrium (LD) and largely tags the H1 and H2 haplotypes, called haplotype tagging SNPs (htSNPs) [20]. Six htSNPs were identified, including 5 htSNPs that represent the intra-H1 variation (rs1467967, rs242557, rs3785883, rs2471738, and rs7521) and a 238-bp insertion/deletion polymorphism within intron 9 (del-In9) (Haplotype H1c: rs1467967 = A, rs242557 = A, rs3785883 = G, rs2471738 = T, del-In9 = ins and rs7521 = G) [20, 21]. The insertion of the del-In9 tags H1 haplotype and the deletion tags H2 haplotype. So, the available data of del-In9 were included into the H2 group to be further analyzed. Two htSNPs were the promoter polymorphisms (rs1467967 and rs242557) and three were intronic (rs3785883, rs2471738, and rs7521) [22]. Additionally, the Q7R (rs62063857) polymorphism of the Saitohin gene (*STH*), nested in intron 9 of the *MAPT*, is in complete LD with the extended H1/H2 haplotype [23, 24]. The Q and R alleles were in LD with H1 and H2 haplotypes, respectively [23, 24]. Unfortunately, some studies showed obvious association of *MAPT* variants with neurodegenerative diseases, while others showed none. The results of the related studies were inconsistent.

Therefore, we performed a meta-analysis by pooling the whole related data from previously studies to reach a more precise estimate of the relationship between variants in *MAPT* and their risk on neurodegenerative diseases.

## RESULTS

**Figure 1:**
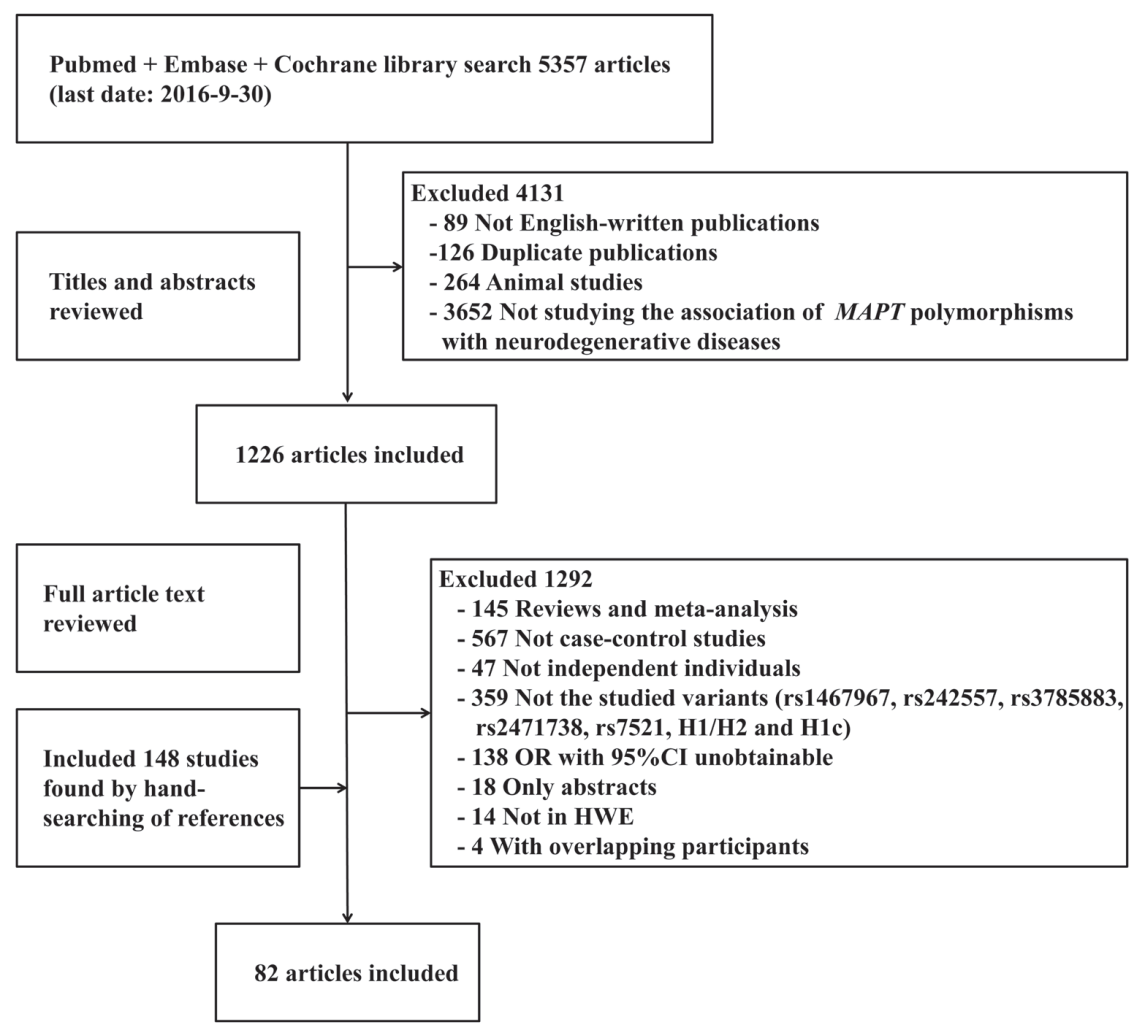
Flow diagram of the study selection process Abbreviations: OR, odds ratio; CI, confidence interval; HWE, Hardy-Weinberg equilibrium.

**Figure 2:**
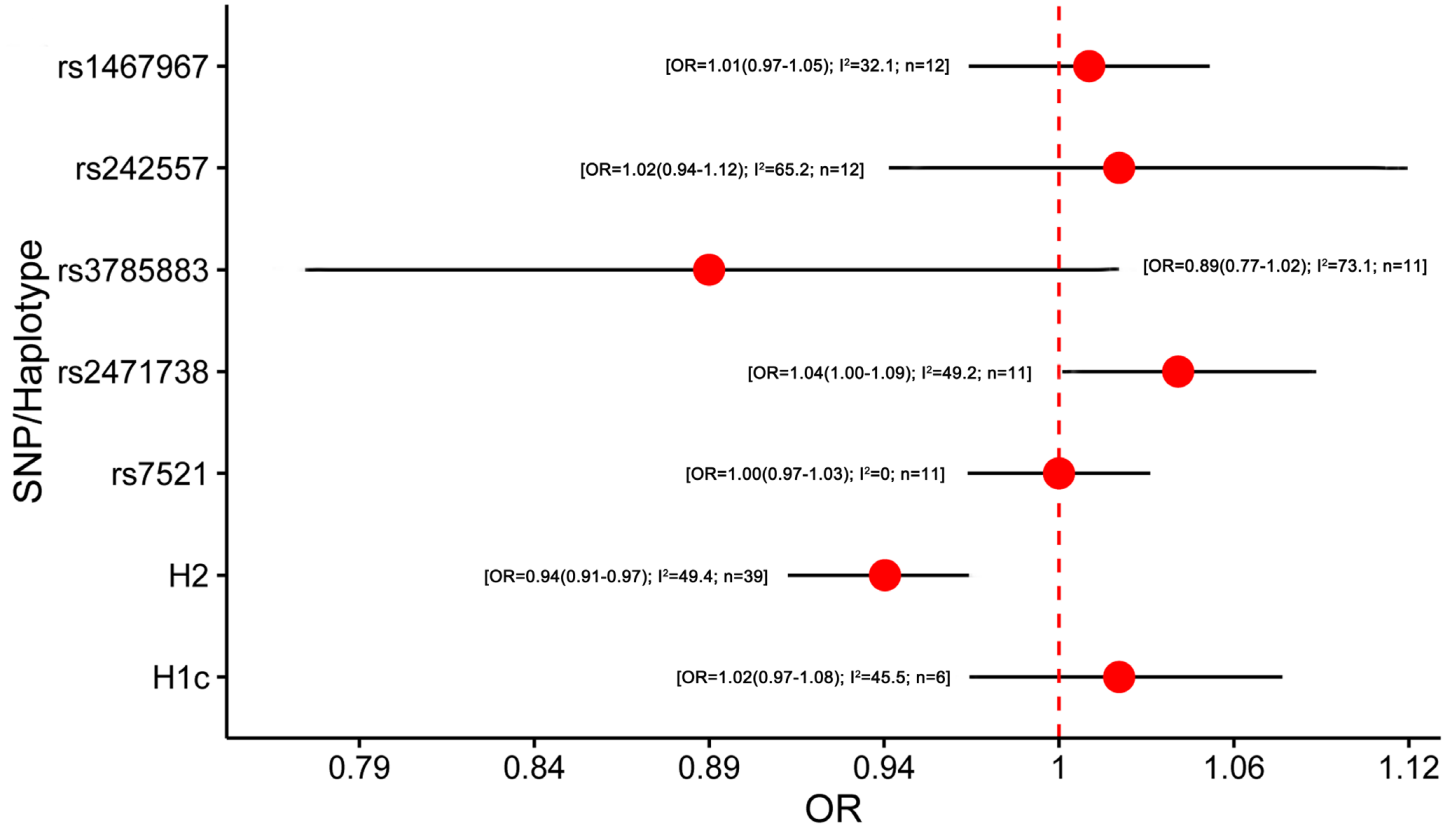
Results of the meta-analysis for five htSNPs, H2 haplotype and H1c subhaplotype in Alzheimer's disease Abbreviations: OR, odds ratio; SNP, single nucleotide polymorphism; I^2^, the heterogeneity calculated by the Cochran Q test; n, the number of included studies.

**Figure 3:**
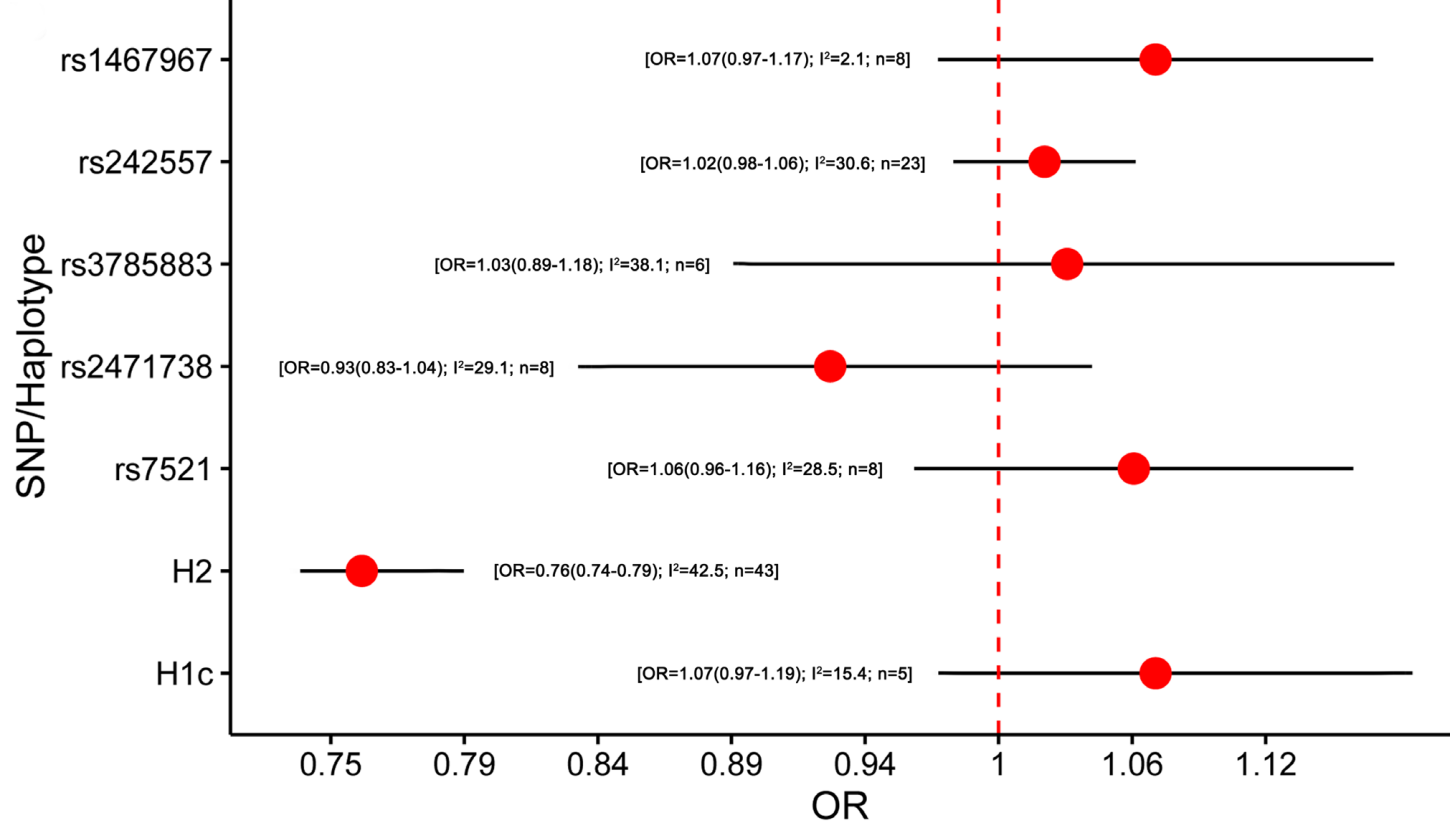
Results of the meta-analysis for five htSNPs, H2 haplotype and H1c subhaplotype in Parkinson's disease Abbreviations: OR, odds ratio; SNP, single nucleotide polymorphism; I^2^, the heterogeneity calculated by the Cochran Q test; n, the number of included studies.

**Figure 4:**
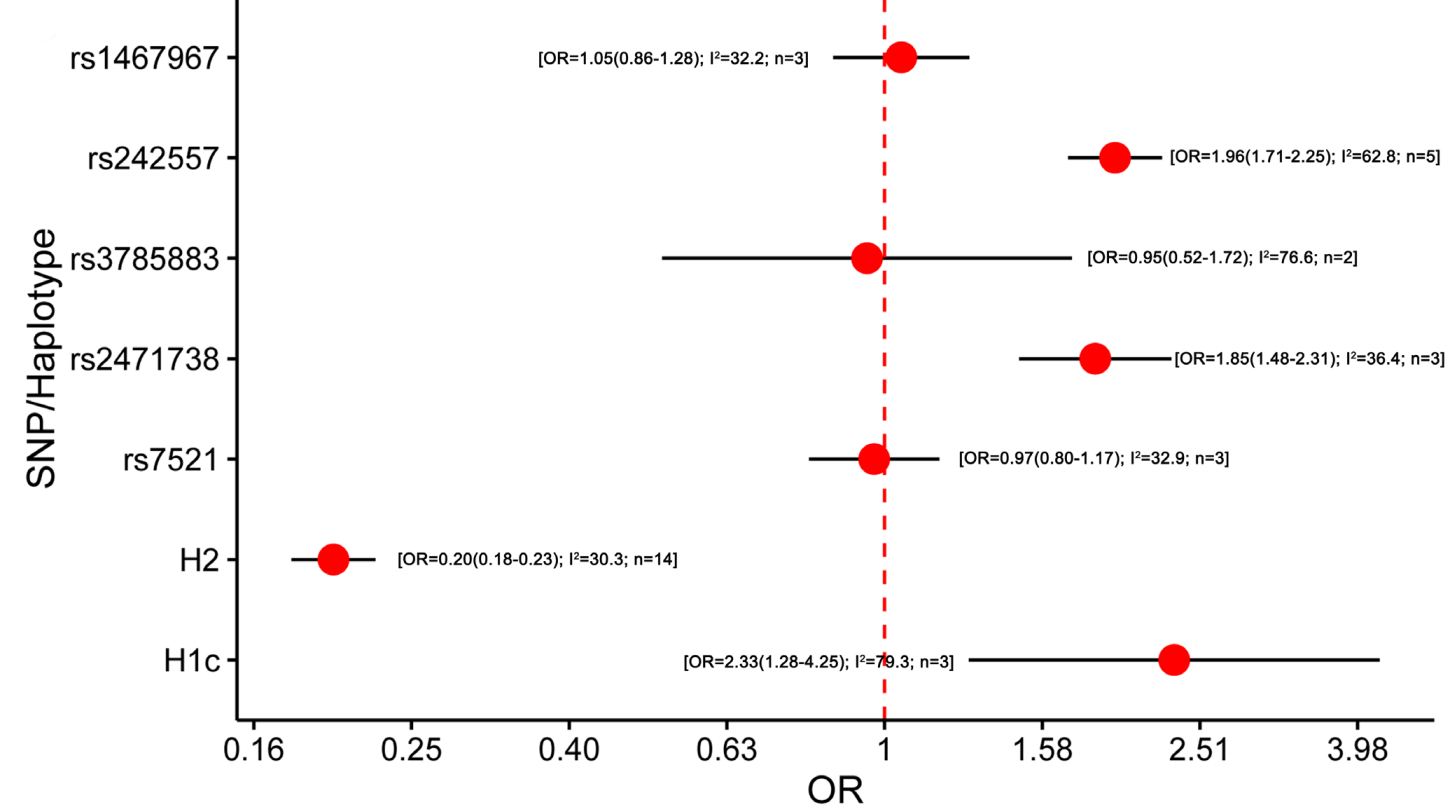
Results of the meta-analysis for five htSNPs, H2 haplotype and H1c subhaplotype in progressive supranuclear palsy Abbreviations: OR, odds ratio; SNP, single nucleotide polymorphism; I^2^, the heterogeneity calculated by the Cochran Q test; n, the number of included studies.

**Figure 5:**
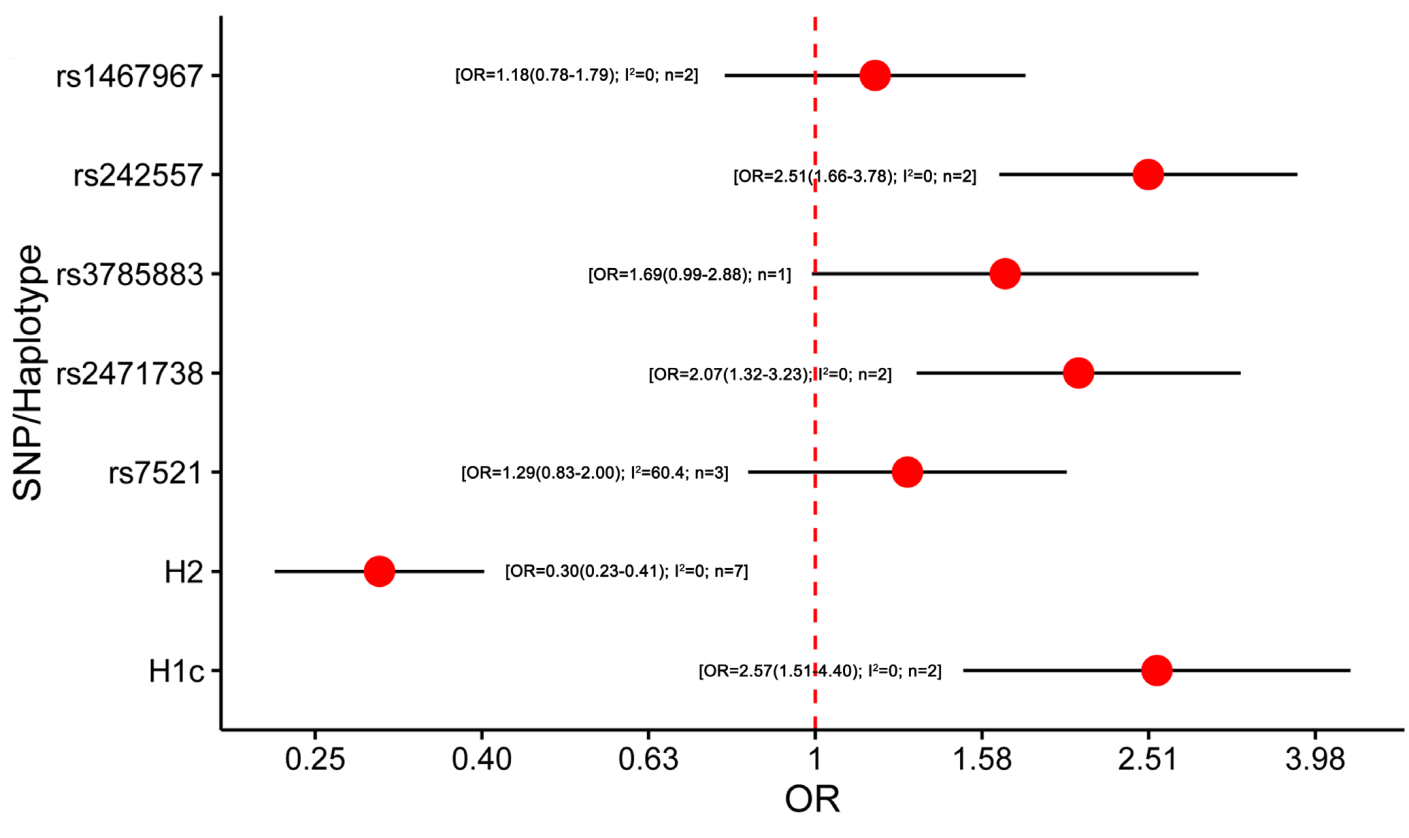
Results of the meta-analysis for five htSNPs, H2 haplotype and H1c subhaplotype in corticobasal degeneration Abbreviations: OR, odds ratio; SNP, single nucleotide polymorphism; I^2^, the heterogeneity calculated by the Cochran Q test; n, the number of included studies.

5357 studies were retrieved through the literature search, and 1226 papers of these were identified as potentially meeting the inclusion criteria after reviewing the titles and abstracts. Besides, we included 148 studies by manual searching. After further reviewing the full text, 82 studies were identified eligible (Figure 1). The results of meta-analysis on the association between variants in *MAPT* and neurodegenerative diseases were shown in Figure 2–5.

### Alzheimer's disease

A total of 37 studies were included in the meta-analysis of *MAPT* polymorphism in AD [4–6, 20, 25–57] (Supplementary Table 2). Among them, 34 studies were conducted in Caucasian populations, and 3 studies were performed in Asian populations. Two studies did not state the diagnostic criteria of AD, and 15 studies did not state whether the polymorphisms were in Hardy-Weinberg equilibrium (HWE). AD cases were diagnosed mainly using the criteria of the National Institute of Neurological and Communication Disorders and Stroke-Alzheimer Disease and Related Disorders Association (NINCDS-ADRDA) [58], or confirmed by autopsy. Notably, our meta-analysis showed that the minor allele (T allele) within rs2471738 was mildly associated with an increased risk of AD (odds ratio (OR) = 1.04, 95% confidence interval (CI) = 1.00 - 1.09) and H2 haplotype might be a protective factor for AD (OR = 0.94, 95% CI = 0.91 - 0.97) (Figure 2; Supplementary Table 8). No associations were found in rs1467967, rs242557, rs3785883, rs7521 and H1c for AD (OR = 1.01, 95% CI = 0.97 -1.05; OR = 1.02, 95% CI = 0.94 -1.12; OR = 0.89, 95% CI = 0.77 -1.02; OR = 1.00, 95% CI = 0.97 -1.03; OR = 1.02, 95% CI = 0.97 -1.08; respectively) (Figure 2; Supplementary Table 8). Due to the ethnic heterogeneity, we performed the subgroup analyses by ethnicity. Interestingly, the minor allele (A allele) within rs3785883 was a protective factor for AD risk in Caucasian (OR = 0.87, 95% CI = 0.76 -1.00) (Supplementary Table 9). This result was calculated by the random-effects model because the heterogeneity existed in the included studies of rs3785883 in Caucasian (I^2^ = 75.2). The heterogeneity was reduced to 56.2% and the pooled effect was changed into negative (OR = 0.94, 95% CI = 0.84 - 1.05) when one single study was excluded [53]. We speculated that the influence of this study maybe result from the origin of the Spanish which was different from other Caucasian populations. Publication bias was assessed by Egger test, and only the subgroup of rs3785883 in Caucasian that showed little publication bias (P _Egger_ = 0.0499) (Supplementary Table 9). Using the trim and fill method to account for the bias did not influence the summary estimate for rs3785883 in Caucasian [59].

### Parkinson's disease

A total of 32 studies were included in the meta-analysis of *MAPT* polymorphism in PD [7, 8, 36, 39, 43, 53, 60–85] (Supplementary Table 3). Among them, 27 studies were conducted in Caucasian populations, 3 studies were performed in Asian populations, and one study was conducted in African populations. Besides, one study included both Caucasian and Asian populations [66]. Six studies lacked diagnostic criteria for PD. Seven studies did not state whether the polymorphisms were within the range of HWE. Most included studies were based on the UK Parkinson's Disease Society Brain Bank clinical diagnostic criteria (UKPDBB) or the extended [86–88], or autopsy. Remarkable, our meta-analysis found that H2 haplotype might be a protective factor for PD (OR = 0.76, 95% CI = 0.74 - 0.79) (Figure 3; Supplementary Table 11). Then we performed the subgroup analyses by ethnicity. Similarly, H2 haplotype showed protective effect on PD in Caucasian subgroup (OR = 0.76, 95% CI = 0.74 - 0.79) (Supplementary Table 12). Additionally, the minor allele within rs242557 (A allele) and rs7521 (A allele) were mildly associated with an increased risk of PD in Caucasians (OR = 1.06, 95% CI = 1.01 - 1.12; OR = 1.11, 95% CI = 1.00 - 1.23, respectively) (Supplementary Table 12). No heterogeneity was existed in each subgroup. The data above were calculated by the fixed-effects model. In addition, based on the results from the Egger test, there was no publication bias.

### Progressive supranuclear palsy

Twelves studies were included in the meta-analysis of *MAPT* polymorphism in PSP [11, 12, 15, 21, 23, 26, 45, 85, 89–92] (Supplementary Table 4). All of the studies were according to standard diagnostic criteria [93, 94]. Five studies did not perform the HWE test. All studies were performed in Caucasians. Notably, our results showed that the minor allele within rs242557 (A allele) and rs2471738 (T allele) may be risk factors for PSP (OR = 1. 96, 95% CI = 1. 71 - 2.25; OR = 1. 85, 95% CI = 1. 48 - 2.31; respectively) (Figure 4; Supplementary Table 14). H2 haplotype showed significantly protective effect on PSP risk (OR = 0.20, 95% CI = 0.18 - 0.23). Additionally, H1c significantly increased the risk of PSP (OR = 2.33, 95% CI = 1.28 - 4.25). The pooled ORs and 95%CIs of three subgroups were calculated by the random-effects model because the heterogeneity evidently existed. For rs242557 subgroup sensitivity analysis, the heterogeneity was reduced to 41.1% and the pooled effect was mildly increased (OR from 1.96 to 2.09) when the stage 2 of the study of Hoglinger et al. was excluded [11]. The majority of cases in the stage 2 of this study were clinically diagnosed and this diagnostic misclassification rate as 12% [11]. Hence, we inferred that the highly diagnostic misclassification rate might affect the result. In addition, for H1c subgroup sensitivity analysis, the heterogeneity was reduced to 28.4% and the pooled effect was mildly increased (OR from 2.33 to 3.17) when one single study conducted in the Spanish was excluded [12]. However, the heterogeneity cannot be further explained for the rs3785883 subgroup, maybe due to too few studies included. In conclusion, heterogeneity and sensitivity analyses showed that the exclusion of the related study did not influence the statistical significances of factors. In addition, no publication bias was found.

### Corticobasal degeneration

Six studies were included in the meta-analysis of *MAPT* polymorphism in CBD [12, 13, 15, 21, 85, 95] (Supplementary Table 5). Only one study lacked the diagnostic criteria for CBD [15], while two studies did not analyze the HWE [13, 85]. All of the included studies were conducted in Caucasian populations. Remarkably, our meta-analysis showed that the minor allele within rs242557 (A allele) and rs2471738 (T allele) may increase the risk of CBD (OR = 2.51, 95%CI = 1. 66 -3.78; OR = 2.07, 95%CI = 1. 32 -3.23; respectively). Notably, H2 haplotype showed protective effect on CBD (OR = 0.30, 95% CI = 0.23 - 0.41), whereas H1c may be risk factor for CBD (OR = 2.57, 95%CI = 1.51 - 4.40) (Figure 5; Supplementary Table 15). The heterogeneity among included studies only existed in rs7521 subgroup. For rs7521 subgroup sensitivity analysis, the heterogeneity was reduced to 0% when one single study which did not report the HWE test was excluded [13]. However, heterogeneity and sensitivity analyses showed that the exclusion of the related study did not influence the statistical significances.

### Frontotemporal dementia

**Figure 6:**
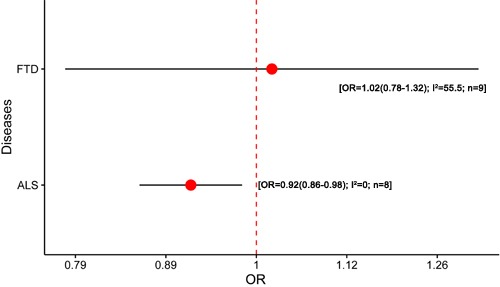
Results of the meta-analysis for H2 haplotype in FTD and ALS Abbreviations: OR, odds ratio; FTD, frontotemporal dementia; ALS, amyotrophic lateral sclerosis; I^2^, the heterogeneity calculated by the Cochran Q test; n, the number of included studies.

Seven studies were included in the meta-analysis of *MAPT* polymorphism in FTD [14–16, 20, 35, 43, 96, 97] (Supplementary Table 6). All studies were conducted in Caucasians. All of the studies were according to the Lund-Manchester criteria or the extended [98, 99]. Three studies did not perform the HWE test [14, 16, 20]. All studies included were only explored the association between *MAPT* haplotypes and FTD. However, the results showed that H2 haplotype was not associated with FTD risk (OR = 1.02, 95% CI = 0.78 - 1.32) (Figure 6; Supplementary Table 16). Mild heterogeneity was existed (I^2^ = 55.5) and reduced to 42.5% when the three studies which did not report the HWE test was excluded. But the exclusion of the related study did not influence the statistical significances.

### Amyotrophic lateral sclerosis

Two studies were included in the meta-analysis of *MAPT* polymorphism in ALS [16, 17] (Supplementary Table 7). All studies were conducted in Caucasians and all cases were diagnosed according to El Escarol criteria for ALS. One study had not performed the HWE test [16]. Like FTD, the studies included were only restricted to the association between *MAPT* haplotypes and ALS. Notably, the H2 haplotype showed mildly protective effect on ALS risk (OR = 0.92, 95% CI = 0.86 - 0.98, I^2^ = 0) (Figure 6; Supplementary Table 17). No heterogeneity was found (I^2^ = 0).

## DISCUSSION

Our findings provide confirmatory evidence that multiple variants in distinct regions in *MAPT* are associated with different neurodegenerative diseases. The minor alleles within rs1467967 and rs242557 are not all the same in different populations (Supplementary Table 1). For uniformity, we all choose the same allele (the minor allele in Caucasians) in subgroups divided by populations for accuracy.

AD is histopathologically characterized by two neuropathological hallmarks: neuritic plaques primarily composed of extracellular Amyloid-β (Aβ) deposits and intracellular neurofibrillary tangles (NFTs) composed of hyper-phosphorylated tau protein [100]. Obviously, tau protein, the *MAPT* gene encoded, plays an essential role in the pathogenesis of AD. It participates in the forming of NFTs. However, the genetic association between *MAPT* variants and AD risk has been inconsistent. Our results showed that rs2471738, rs3785883 and H2 haplotype were associated with AD. Several studies found that the pathogenesis of AD conferred by *MAPT* variants might be increasing levels of total or/and 4-repeat (4R) tau [6, 48, 101]. The T allele at rs2471738 has been associated with increased AD risk. But this SNP lies within a region of intron 9, and does not appear to interrupt the splice sites. This suggests that perhaps rs2471738 is not functional, but is in LD with other functional variants [4, 50]. Notably, the A allele at rs3785883 was found with a decreased risk of AD only in Caucasians. However, after the heterogeneity and sensitivity analysis, the association changed into negative when one study was excluded [53]. Hence, the protective effect of the A allele at rs3785883 on AD development in Caucasians is unreliable and awaits further investigation. Remarkably, both the two included studies showed that the A allele at rs242557 was associated with a decreased risk of AD in Asian [55, 56]. When pooling together, our result showed no association. So further studies are needed to verify this association since our result was only based on two studies.

PD is the second most common neurodegenerative disease following AD. Recently, some genome-wide association studies (GWAS) have provided robust evidence for the genetic association of *MAPT* with PD [7, 8, 73]. Likewise, our pooled analysis showed a robust association between *MAPT* and PD. We found that rs242557, rs7521 and H2 haplotype were associated with PD in Caucasian. Besides, *MAPT* variants influence the susceptibility of PD risk mainly by affecting the levels of total or 4R tau [8]. Then the tau would interact with α-synuclein, which forms the Lewy body in PD [102]. This process can promote the fibrillization of both tau and α-synuclein, and drive the formation of pathological inclusions in PD [103]. Rs242557 in the promoter of *MAPT* has been identified as a functional variant, which could affect the expression or splicing of *MAPT*, acting as cis-factors [83, 104]. Therefore, we speculate that rs242557-A allele can increase the brain tau levels in PD patients and thus raise the risk of PD susceptibility in Caucasians. For rs7521, the A allele could lower the age at onset (AAO) of PD and in reverse the G allele delays the AAO [70]. This finding is identical to our result that the A allele at rs7521 could increase the risk of PD in Caucasians. The mechanism may be that rs7521 is in LD with other risky SNPs [70]. We confirmed that H2 haplotype could decrease the risk of PD in Caucasians. However, only one study in Asian and one in African were included, and so the association of *MAPT* haplotype with PD in non-Caucasians awaits further investigation.

PSP and CBD are two parkinsonian syndromes characterized by deposits of neurofibrillary tangles in the brain, which are mainly composed of 4R *MAPT* protein isoforms [105]. In healthy adult brain, the levels of 3R-tau and 4R-tau are approximately equal. 4R-tau were nearly three times faster than 3R-tau for the rates of promoting to microtubule assemble to form the NTFs [3]. Probably, the two disorders are genetically related diseases and share a similar cause that involves tau dysfunction [12, 95]. However, PSP and CBD are usually considered sporadic disorders, so the genetics of the two disorders has been seldom researched [89, 95]. Hence, our meta-analysis is essential for identifying the genetics of PSP and CBD by pooling the limited studies. We confirmed that rs242557, rs2471738 and H1c subhaplotype could increase the risk of PSP and CBD, while H2 haplotype decrease. What's more, these results were further confirmed that PSP and CBD might share a similar genetic background. Similarly, the mechanisms for the associations of rs242557, rs2471738, H2 and H1c with PSP and CBD may be due to influence the levels of tau, especially the 4R tau. However, only two studies were included in the subgroup of rs242557, rs2471738 and H1c in CBD. So, these results should be treated with cautious and more related studies are needed in the further.

FTD is a form of presenile dementia characterized clinically by behavioral and personality changes, mutism and decline of memory later in the disease [96], which neuropathologically affecting the frontal and/or temporal lobes. Many studies confirmed that mutations in the *MAPT* gene had been detected in autosomal dominant FTD with parkinsonism (FTDP). In addition, the accumulation of aberrant tau protein was found in FTD. These findings suggested that *MAPT* may be a genetic risk factor for FTD. However, our meta-analysis was failed to find the *MAPT* haplotypes had been associated with FTD. We suggest that more studies on other variants in *MAPT* are needed to probe the association and also in other populations.

ALS is a rarely progressive neurodegenerative disorder, but is the most common motor neuron disease in adult-onset. ALS is characterized by the simultaneous deficits of upper and lower motor neurons, which lead to muscle atrophy and paralysis [106]. The hyperphosphorylated and aggregated tau have been found in ALS, while the association was uncertain. We pooled that presently limited studies and found that H2 haplotype may be a mildly protective factor for ALS.

Our meta-analyses provide confirmatory evidence that multiple variants in *MAPT* are associated with neurodegenerative diseases risk. But there are several limits in our study. One limitation of our study is that the present studies were only limited to Caucasian populations in PSP, CBD, FTD and ALS, to our knowledge. What is more, in FTD and ALS, the studies were only limited to H1/H2 haplotype. Therefore, more studies are needed in the future, particularly those which performed in different populations and SNPs. Another limitation is different diagnostic criteria across studies. The accuracy of clinical diagnostic criteria is lower than pathological diagnostic criteria. For example, we assumed that about 6% of the patients had PSP but might be clinically diagnosed as PD by mistake [88]. Furthermore, we divided the data into subgroups by different criteria. But there was no difference across the pathological and clinical diagnostic subgroups.

In conclusion, this is the first comprehensive meta-analysis and systematic review which focused on the association between *MAPT* polymorphisms and neurodegenerative diseases so far. Our results robustly confirm the susceptibility role of *MAPT* in neurodegenerative diseases in a large meta-analysis, using highly informative htSNPs which capture 95% of *MAPT* haplotype diversity. Here we analyze these variants in *MAPT* and their association with neurodegenerative diseases as a step toward determining the precise mechanisms of genetic susceptibility for these group diseases. Significantly, this will have important implications for identifying potential therapeutic targets.

## MATERIALS AND METHODS

### Identification and selection of relevant studies

In order to identify the association between the *MAPT* polymorphism with neurodegenerative diseases, we conducted a literature search in MEDLINE, EMBASE and the Cochrane library up to September 2016. We used the key search terms including “microtubule-associated protein tau”, “*MAPT*”, “saitohin”, “STH”, “polymorphism”, “Alzheimer's disease”, “Parkinson's disease”, “progressive supranuclear palsy”, “corticobasal degeneration”, “frontotemporal dementia”, “amyotrophic lateral sclerosis” and “neurodegenerative disease”, combined with Boolean operators as appropriate. Additional studies were obtained from the reference lists of relevant original studies or review articles.

We assessed all studies if they meet the following criteria: (1) Evaluation of an association between *MAPT* polymorphisms and neurodegenerative diseases; (2) Case-control studies design; (3) Available allelic frequency for *MAPT* rs1467967, rs242557, rs3785883, rs2471738, rs7521, H2 and H1c haplotypes; Data of allelic frequency for estimating the OR with corresponding 95% CI were available in the report or could be calculated. We exclude the study, which the genotype frequency in the control group was not in HWE. Additionally, when two or more studies had overlapping participants, only the one with larger sample size was included.

### Data extraction and quality assessment

Two reviewers independently read the studies and extracted data according to predefined criteria. Data of allelic frequency and its OR with corresponding 95% CI were extracted. Besides, the following information was extracted: author, publication year, state, ethnicity, diagnostic criteria, type of neurodegenerative diseases, sample size, mean age, the percentage of female, minor allele frequency, and the HWE test in each control group. The quality of studies was assessed with the Newcastle-Ottawa quality Scale (NOS): 1) the Selection; 2) the Comparability; 3) the Exposure [107]. Studies with a score of at least seven points were considered to be high quality.

### Statistical analysis

To determine the strength of associations between individual *MAPT* polymorphism and neurodegenerative disease, we calculated a pooled OR and 95 % CI using R software. The OR and 95 % CI were evaluated by comparison in minor and major allele frequency using the Pearson χ^2^ test or Fisher's exact test. We used the random-effects and fixed-effects model to calculate the subgroup and pooled ORs with 95% CIs in the presence and absence of heterogeneity, respectively. We assessed the heterogeneity by the Cochran Q and I^2^ (*P* > 0.10 or I^2^ < 50%, mean a lack of heterogeneity). Publication bias was detected by Egger's test (*P* > 0.05, mean no publication bias) [108]. When k ≥ 10, we calculate the P value of Egger's test using R software (k, means the number of studies). We used the trim and fill method to adjust the statistically significant publication bias [59]. In addition, as ethnic-specific susceptibility variants exist, we reanalyzed the data according to different races (Caucasian and Asian).

## SUPPLEMENTARY MATERIALS FIGURES AND TABLES



## Abbreviations

*MAPT*: microtubule-associated protein tau
AD: Alzheimer's disease
PD: Parkinson's disease
PSP: progressive supranuclear palsy
CBD: corticobasal degeneration
FTD: frontotemporal dementia
ALS: amyotrophic lateral sclerosis
SNPs: single-nucleotide polymorphisms
htSNPs: haplotype tagging SNPs
LD: complete linkage disequilibrium
del-In9: a 238-bp insertion/deletion polymorphism within intron 9
*STH*: Saitohin gene
HWE: Hardy-Weinberg equilibrium
NINCDS-ADRDA: the diagnostic criteria of the National Institute of Neurological and Communication Disorders and Stroke-Alzheimer Disease and Related Disorders Association
OR: odds ratio
CI: confidence interval
UKPDBB: the UK Parkinson's Disease Society Brain Bank clinical diagnostic criteria
Aβ: Amyloid-β
4R: 4-repeat
GWAS: genome-wide association studies
AAO: age at onset
FTDP: FTD with parkinsonism
NOS: Newcastle-Ottawa quality Scale.
