# Supplementary material for: Meta-analysis of the association between variants in MAPT and neurodegenerative diseases
Source: Oncotarget. 2017 Mar 29;8(27):44994–5007. doi: 10.18632/oncotarget.16690 (PMC5546535; doi:10.18632/oncotarget.16690)

Meta-analysis of the association between variants in *MAPT* and neurodegenerative diseases

Supplementary Material

Supplementary Table 1 The 5 htSNPs and del-In9 used for the meta-analysis of *MAPT*

SNP	Chromosomal location	dbSNP ID	Location in *MAPT*	Minor allele
1	41342006	rs1467967	5′ of exon 1	G[a] (A[b])
2	41375573	rs242557	5′ of exon 1	A[c] (G[d])
3	41410269	rs3785883	intron 3	A
4	41431900	rs2471738	intron 9	T
5	41442488	del-In9	intron 9	H2
6	41461242	rs7521	3′ of exon 14	A

The above information was obtained from HapMap (http://www.hapmap.org/).

Abbreviation: htSNPs, haplotype tagging single nucleotide polymorphisms.

[a] The minor allele within rs1467967 in Caucasian

[b] The minor allele within rs1467967 in Chinese and Japanese

[c] The minor allele within rs242557 in Caucasian and Japanese

[d] The minor allele within rs242557 in Chinese

Supplementary Table 2 Characteristics of included studies in AD

Author/ Year	State	Ethnicity	Diagnostic criteria	Cases			Controls			SNP/ Haplotype	HWE (Y/N)	NOS	PMID
				N	Mean age (SD/range)	Female %	N	Mean age (SD/range)	Female %				
Myers 2005(US)	US	Caucasians	Autopsy confirmed	181	81 (66–97) [a]	55.00%	131	81 (65–99)	51.00%	rs1467967	-	7	16000317
Myers 2005(UK)	UK	Caucasians	Autopsy confirmed	179	81 (65–96) [a]	66.00%	121	78 (65–100)	51.00%	rs1467967	-	7	16000317
Myers 2007	US	Caucasians	Autopsy confirmed	296	83 (65–102) [a]	55.00%	128	80 (65–102)	44.00%	rs1467967	-	7	17174556
Laws 2007	Germany	Caucasians	NINCDS-ADRDA	433	69.3 (9.4) [b]	59.00%	279	67.2 (11.6)	57.00%	rs1467967	Y	9	17179995
Mukherjee 2007	US	Caucasians	NINCDS-ADRDA	361	-	-	358	-	-	rs1467967	Y	7	17266761
Abraham 2009	UK	Caucasians	NINCDS-ADRDA	982	75.84 (6.79) [b]	71.00%	1153	76.53 (6.33)	62.00%	rs1467967	Y	8	19308965
Feulner 2010	Germany	Caucasians	NINCDS-ADRDA	491	72.2 [c]	56.62%	479	39.5	46.35%	rs1467967	-	7	19125160

Cousin 2011	France	Caucasians	NINCDS-ADRDA	420	64.9 (9.9) [b]	-	456	66.2 (10.8)	-	rs1467967	Y	7	19889475
Seto-Salvia 2011	Spain	Caucasian	NINCDS-ADRDA	164	77.0 (5.5) [b]	69.50%	374	81.3 (6.9)	68.20%	rs1467967	Y	7	21403021
Elias-Sonnenschein 2013	Finland	Caucasian	NINCDS-ADRDA	869	69.8 (8.2) [b]	67.00%	685	69.1 (6.2)	60.00%	rs1467967	Y	8	23573206
Chang 2014	Taiwan	Asian	NINCDS-ADRDA	108	70.7 (8.52) [d]	45.40%	108	70.7 (8.69)	45.40%	rs1467967	Y	8	24923570
Allen 2014	US	Caucasian	NINCDS-ADRDA, autopsy confirmed	8978	-	-	10373	-	-	rs1467967	Y	7	25324900
Myers 2005(US)	US	Caucasian	Autopsy confirmed	181	81 (66–97) [a]	55.00%	131	81 (65–99)	51.00%	rs242557	-	7	16000317
Myers 2005(UK)	UK	Caucasian	Autopsy confirmed	179	81 (65–96) [a]	66.00%	121	78 (65–100)	51.00%	rs242557	-	7	16000317
Myers 2007	US	Caucasians	Autopsy confirmed	296	83 (65–102) [a]	55.00%	128	80 (65–102)	44.00%	rs242557	-	7	17174556
Laws 2007	Germany	Caucasians	NINCDS-ADRDA	433	69.3 (9.4) [b]	59.00%	279	67.2 (11.6)	57.00%	rs242557	Y	9	17179995
Mukherjee 2007	US	Caucasians	NINCDS-ADRDA	361	-	-	358	-	-	rs242557	Y	7	17266761
Abraham 2009	UK	Caucasians	NINCDS-ADRDA	979	75.84 (6.79) [b]	71.00%	1139	76.53 (6.33)	62.00%	rs242557	Y	8	19308965
Feulner 2010	Germany	Caucasians	NINCDS-ADRDA	491	72.2 [c]	56.62%	479	39.5	46.35%	rs242557	-	7	19125160
Cousin 2011	France	Caucasians	NINCDS-ADRDA	417	64.9 (9.9) [b]	-	455	66.2 (10.8)	-	rs242557	Y	7	19889475
Seto-Salvia 2011	Spain	Caucasian	NINCDS-ADRDA	164	77.0 (5.5) [b]	69.50%	374	81.3 (6.9)	68.20%	rs242557	Y	7	21403021
Liu 2013	China	Asian	NINCDS-ADRDA	796	75.28 (6.57) [b]	49.75%	796	74.81 (6.96)	48.74%	rs242557	Y	9	23116876
Chang 2014	Taiwan	Asian	NINCDS-ADRDA	108	70.7 (8.52) [d]	45.40%	108	70.7 (8.69)	45.40%	rs242557	Y	8	24923570
Allen 2014	US	Caucasian	NINCDS-ADRDA, autopsy confirmed	8507	-	-	9835	-	-	rs242557	Y/N [e]	7	25324900
Myers 2005(US)	US	Caucasian	Autopsy confirmed	181	81 (66–97) [a]	55.00%	131	81 (65–99)	51.00%	rs3785883	-	7	16000317
Myers 2005(UK)	UK	Caucasian	Autopsy confirmed	179	81 (65–96) [a]	66.00%	121	78 (65–100)	51.00%	rs3785883	-	7	16000317
Myers 2007	US	Caucasians	Autopsy confirmed	296	83 (65–102) [a]	55.00%	128	80 (65–102)	44.00%	rs3785883	-	7	17174556
Laws 2007	Germany	Caucasians	NINCDS-ADRDA	433	69.3 (9.4) [b]	59.00%	279	67.2 (11.6)	57.00%	rs3785883	Y	9	17179995
Mukherjee 2007	US	Caucasians	NINCDS-ADRDA	361	-	-	358	-	-	rs3785883	Y	7	17266761
Abraham 2009	UK	Caucasians	NINCDS-ADRDA	967	75.84 (6.79) [b]	71.00%	1139	76.53 (6.33)	62.00%	rs3785883	Y	8	19308965
Feulner 2010	Germany	Caucasians	NINCDS-ADRDA	491	72.2 [c]	56.62%	479	39.5	46.35%	rs3785883	-	7	19125160
Cousin 2011	France	Caucasians	NINCDS-ADRDA	422	64.9 (9.9) [b]	-	451	66.2 (10.8)	-	rs3785883	Y	7	19889475
Seto-Salvia 2011	Spain	Caucasian	NINCDS-ADRDA	164	77.0 (5.5) [b]	69.50%	374	81.3 (6.9)	68.20%	rs3785883	Y	7	21403021
Chang 2014	Taiwan	Asian	NINCDS-ADRDA	108	70.7 (8.52) [d]	45.40%	108	70.7 (8.69)	45.40%	rs3785883	Y	8	24923570
Allen 2014	US	Caucasian	NINCDS-ADRDA, autopsy confirmed	9351	-	-	11083	-	-	rs3785883	Y	7	25324900

Myers 2005(US)	US	Caucasian	Autopsy confirmed	181	81 (66–97) [a]	55.00%	131	81 (65–99)	51.00%	rs2471738	-	7	16000317
Myers 2005(UK)	UK	Caucasian	Autopsy confirmed	179	81 (65–96) [a]	66.00%	121	78 (65–100)	51.00%	rs2471738	-	7	16000317
Myers 2007	US	Caucasians	Autopsy confirmed	296	83 (65–102) [a]	55.00%	128	80 (65–102)	44.00%	rs2471738	-	7	17174556
Laws 2007	Germany	Caucasians	NINCDS-ADRDA	433	69.3 (9.4) [b]	59.00%	279	67.2 (11.6)	57.00%	rs2471738	Y	9	17179995
Mukherjee 2007	US	Caucasians	NINCDS-ADRDA	361	-	-	358	-	-	rs2471738	Y	7	17266761
Mateo 2008	Spain	Caucasians	NINCDS-ADRDA	293	72.1 (8.2) [b]	65.00%	396	81.0 (7.6)	67.00%	rs2471738	Y	9	18319590
Abraham 2009	UK	Caucasians	NINCDS-ADRDA	970	75.84 (6.79) [b]	71.00%	1125	76.53 (6.33)	62.00%	rs2471738	Y	8	19308965
Cousin 2011	France	Caucasians	NINCDS-ADRDA	423	64.9 (9.9) [b]	-	454	66.2 (10.8)	-	rs2471738	Y	7	19889475
Seto-Salvia 2011	Spain	Caucasian	NINCDS-ADRDA	164	77.0 (5.5) [b]	69.50%	374	81.3 (6.9)	68.20%	rs2471738	Y	7	21403021
Chang 2014	Taiwan	Asian	NINCDS-ADRDA	108	70.7 (8.52) [d]	45.40%	108	70.7 (8.69)	45.40%	rs2471738	Y	8	24923570
Allen 2014	US	Caucasian	NINCDS-ADRDA, autopsy confirmed	8922	-	-	10541	-	-	rs2471738	Y	7	25324900
Myers 2005(US)	US	Caucasian	Autopsy confirmed	181	81 (66–97) [a]	55.00%	131	81 (65–99)	51.00%	rs7521	-	7	16000317
Myers 2005(UK)	UK	Caucasian	Autopsy confirmed	179	81 (65–96) [a]	66.00%	121	78 (65–100)	51.00%	rs7521	-	7	16000317
Myers 2007	US	Caucasians	Autopsy confirmed	296	83 (65–102) [a]	55.00%	128	80 (65–102)	44.00%	rs7521	-	7	17174556
Laws 2007	Germany	Caucasians	NINCDS-ADRDA	433	69.3 (9.4) [b]	59.00%	279	67.2 (11.6)	57.00%	rs7521	Y	9	17179995
Mukherjee 2007	US	Caucasians	NINCDS-ADRDA	361	-	-	358	-	-	rs7521	Y	7	17266761
Abraham 2009	UK	Caucasians	NINCDS-ADRDA	990	75.84 (6.79) [b]	71.00%	1148	76.53 (6.33)	62.00%	rs7521	Y	8	19308965
Cousin 2011	France	Caucasians	NINCDS-ADRDA	420	64.9 (9.9) [b]	-	452	66.2 (10.8)	-	rs7521	Y	7	19889475
Seto-Salvia 2011	Spain	Caucasian	NINCDS-ADRDA	164	77.0 (5.5) [b]	69.50%	374	81.3 (6.9)	68.20%	rs7521	Y	7	21403021
Elias-Sonnenschein 2013	Finland	Caucasian	NINCDS-ADRDA	869	69.8 (8.2) [b]	67.00%	683	69.1 (6.2)	60.00%	rs7521	Y	8	23573206
Chang 2014	Taiwan	Asian	NINCDS-ADRDA	108	70.7 (8.52) [d]	45.40%	108	70.7 (8.69)	45.40%	rs7521	Y	8	24923570
Allen 2014	US	Caucasian	NINCDS-ADRDA, autopsy confirmed	9577	-	-	11250	-	-	rs7521	Y	7	25324900
Crawford 1999	US	Caucasian	NINCDS-ADRDA	65	74.51 (7.14) [d]	47.00%	142	75.15 (7.30)	60.00%	H2	-	7	10465706
Crawford 1999	US	Caucasian	NINCDS-ADRDA	200	72.80 (5.97) [d]	47.00%	142	75.15 (7.30)	60.00%	H2	-	8	10465706
Ezquerra 1999	-	Caucasians	NINCDS-ADRDA	74	65.2 (10.5) [b]	51.35%	195	61.4 (13.9)	51.28%	H2	-	8	10580705
Roks 1999	Netherlands	Caucasians	NINCDS-ADRDA	101	57 (5.0) [b]	-	116	61 (3.3)	-	H2	Y	7	10624829
Lilius 1999	Sweden	Caucasians	NINCDS-ADRDA	175	65 (9) [b]	-	62	73 (12)	-	H2	-	7	10643890
Lilius 1999	Sweden	Caucasians	DSM III-R	94	80 (5) [b]	-	176	88 (4)	-	H2	-	7	10643890
Bullido 2000	Spain	Caucasians	NINCDS-ADRDA	167	69.1 (5.1) [b]	-	194	68.2 (7.4)	-	H2	-	7	10643798

Kwon 2000	US	Caucasian	-	266	70 (9.1) [b]	50.20%	278	74.5 (9.0)	59.30%	H2	Y	7	10771166
Baker 2000	Finland	Caucasian	-	182	-	-	223	-	-	H2	-	6	10793248
Baker 2000	US	Caucasian	-	271	-	-	419	-	-	H2	-	6	10793248
Russ 2001	UK	Caucasian	NINCDS-ADRDA	200	77.5 (7.3) [b]	76.00%	189	80.1 (4)	-	H2	Y	7	11698154
Conrad 2002	-	Caucasian	Autopsy confirmed	51	80.51 [d]	-	30	78.83	-	H2	-	7	12032355
Cook 2002	UK	Caucasian	Autopsy confirmed	203	81.4 (7.8) [a]	65.52%	309	82.1 (3.8)	58.90%	H2	-	8	12402275
Verpillat 2002	France	Caucasian	NINCDS-ADRDA	499	63.8 (9.7) [b]	62.00%	402	66.6 (10.2)	52.00%	H2	Y	8	12447938
Streffer 2003(Greece)	Greece	Caucasian	NINCDS-ADRDA	134	71.61 (6.9) [b]	-	52	70.0 (6.3)	-	H2	Y	7	12588928
Streffer 2003(Switzerland)	Switzerland	Caucasian	NINCDS-ADRDA	91	71.61 (6.9) [b]	-	92	70.0 (6.3)	-	H2	Y	7	12588928
Combarros 2003	Spain	Caucasian	NINCDS-ADRDA	315	71.9 (8.8) [b]	70.00%	307	80.5 (7.7)	72.00%	H2	Y	8	12826738
Clark 2003	US	Caucasian	NINCDS-ADRDA, DSM-III-R	200	81.6 (7.0) [c]	77.50%	458	75.7 (6.0)	64.20%	H2	Y	8	12865131
Oliveria 2003	US	Caucasian	CERAD and NIA-Reagan	903	-	66.67%	320	-	54.38%	H2	Y	7	12875906
Peplonska 2003	Poland	Caucasian	NINCDS-ADRDA	100	71.5 (4.2) [b]	64.00%	100	71.2 (5.9)	79.00%	H2	-	8	12932819
Conrad 2004	Germany	Caucasian	Autopsy confirmed	155	80.9 (6.9) [a]	-	41	76.1 (5.6)	-	H2	-	7	15030402
Seripa 2004(Italy)	Italy	Caucasian	NINCDS-ADRDA	130	65.91 (7.20) [b]	75.00%	105	62.42 (10.55)	48.00%	H2	-	8	15136700
Seripa 2004(US)	US	Caucasian	Autopsy confirmed	117	71.65 (8.80) [b]	64.00%	99	83.75 (8.22)	53.00%	H2	-	8	15136700
Myers 2005(US)	US	Caucasian	Autopsy confirmed	181	81 (66–97) [a]	55.00%	131	81 (65–99)	51.00%	H2	-	7	16000317
Myers 2005(UK)	UK	Caucasian	Autopsy confirmed	179	81 (65–96) [a]	66.00%	121	78 (65–100)	51.00%	H2	-	7	16000317
Johansson 2005	Sweden	Caucasian	NINCDS-ADRDA	398	73 (7.7) [b]	58.30%	186	72 (9.2)	56.50%	H2	Y	7	16909000
Zuo 2006	US	Caucasian	NINCDS-ADRDA	286	69.3 (8.3) [b]	63.29%	197	-	55.84%	H2	Y	7	16603077
Myers 2007	US	Caucasians	Autopsy confirmed	296	83 (65–102) [a]	55.00%	128	80 (65–102)	44.00%	H2	-	7	17174556
Laws 2007	Germany	Caucasians	NINCDS-ADRDA	433	69.3 (9.4) [b]	59.00%	279	67.2 (11.6)	57.00%	H2	Y	9	17179995
Mukherjee 2007	US	Caucasians	NINCDS-ADRDA	361	-	-	358	-	-	H2	Y	7	17266761
Ezquerra 2007	Spain	Caucasians	Autopsy confirmed	10	61.4 (11.6) [b]	70.00%	6	-	66.67%	H2	-	5	17320831
Lin 2008	China	Asian	NINCDS-ADRDA, DSM-IV	280	75.75 (9.68) [b]	75.36%	220	78.49 (8.92)	71.82%	H2	Y	8	18850062
Kaivorinne 2008	Finland.	Caucasian	NINCDS-ADRDA	122	58.2 [d]	55.00%	198	40.6	-	H2	-	7	19091059
Abraham 2009	UK	Caucasians	NINCDS-ADRDA	962	75.84 (6.79) [b]	71.00%	1126	76.53 (6.33)	62.00%	H2	Y	8	19308965
Feulner 2010	Germany	Caucasians	NINCDS-ADRDA	491	72.2 [c]	56.62%	479	39.5	46.35%	H2	-	7	19125160

Cousin 2011	France	Caucasians	NINCDS-ADRDA	412	64.9 (9.9) [b]	-	444	66.2 (10.8)	-	H2	Y	7	19889475
Seto-Salvia 2011	Spain	Caucasian	NINCDS-ADRDA	164	77.0 (5.5) [b]	69.50%	374	81.3 (6.9)	68.20%	H2	Y	7	21403021
Allen 2014	US	Caucasian	NINCDS-ADRDA, autopsy confirmed	9660	-	-	11364	-	-	H2	Y	7	25324900
Pastor 2015	Spain	Caucasian	NINCDS-ADRDA	4327	76.5 (9.3) [b]	69.00%	5950	64.1 (14.8)	62.10%	H2	Y	8	26444794
Myers 2005(US)	US	Caucasian	Autopsy confirmed	181	81 (66–97) [a]	55.00%	131	81 (65–99)	51.00%	H1c	-	7	16000317
Myers 2005(UK)	UK	Caucasian	Autopsy confirmed	179	81 (65–96) [a]	66.00%	121	78 (65–100)	51.00%	H1c	-	7	16000317
Abraham 2009	UK	Caucasian	NINCDS-ADRDA	997	75.84 (6.79) [b]	71.00%	1164	76.53 (6.33)	62.00%	H1c	Y	8	19308965
Cousin 2011	France	Caucasians	NINCDS-ADRDA	423	64.9 (9.9) [b]	-	456	66.2 (10.8)	-	H1c	Y	7	19889475
Seto-Salvia 2011	Spain	Caucasian	NINCDS-ADRDA	164	77.0 (5.5) [b]	69.50%	374	81.3 (6.9)	68.20%	H1c	Y	7	21403021
Allen 2014	US	Caucasian	NINCDS-ADRDA, autopsy confirmed	8507	-	-	9835	-	-	H1c	Y	7	25324900

Abbreviation: N, the number of cases/controls; SD, standard deviation; NOS, Newcastle-Ottawa Scale; PMID, PubMed-Indexed for MEDLINE; US, the United States; UK, the United Kingdom; NINCDS–ADRDA, the criteria of the National Institute of Neurological and Communicative Disorders and Stroke–Alzheimer’s Diseases and Related Disorders Association; DMS-IV, Diagnostic and Statistical Manual of Mental Disorders 4th Edition; DSM III-R, Diagnostic and Statistical Manual of Mental Disorders 3rd Edition; CERAD and NIA-Reagan, the neuropathological criteria of the Consortium to Establish a Registry for Alzheimer’s Disease and National Institute on Aging and the Reagan Institute; HWE, Hardy–Weinberg equilibrium; - , not obtained.

[a] Mean age at death

[b] Mean age at onset

[c] Mean age at examination

[d] Not indicated

[e] This sample was composed of two groups. One in HWE, while one not.

Supplementary Table 3 Characteristics of included studies in PD

Author/ Year	State	Ethnicity	Diagnostic criteria	Cases			Controls			SNP/ Haplotype	HWE (Y/N)	NOS	PMID
				N	Mean age (SD/range)	Female%	N	Mean age (SD/range)	Female %				
Fung 2006	Greece	Caucasian	UKPDBB	100	63.3 (9.6) [b]	41.30%	94	68.3 (12.8)	43.70%	rs1467967	Y	8	17192721
Fung 2006	Finland	Caucasian	UKPDBB	60	61.5 (8.8) [b]	40.80%	86	66.4 (9.2)	63.20%	rs1467967	Y	8	17192721
Fung 2006	Taiwan	Asian	UKPDBB	56	61.7 (10.9) [b]	47.10%	114	59.0 (10.1)	43.80%	rs1467967	Y	8	17192721
Vandrovcova 2009	UK	Caucasian	Autopsy confirmed	324	-	-	180	-	-	rs1467967	Y	7	18162161
Vandrovcova 2009	UK	Caucasian	-	248	-	-	480	-	-	rs1467967	Y	7	18162161
Das 2009	India	Asian	UKPDBB	301	45 (11) [b]	25.00%	243	49 (8)	21.00%	rs1467967	Y	8	19450659

Ezquerra 2011	Spain	Caucasian	Hughes et al., 1992	505	56.6 (10.9) [b]	46.00%	233	68.8 (9)	48.00%	rs1467967	-	8	19879020
Seto-Salvia 2011	Spain	Caucasian	Hughes et al., 1992	202	58.1 (10.8) [b]	46.10%	374	81.3 (6.9)	68.20%	rs1467967	Y	7	21403021
Fung 2006	Greece	Caucasian	UKPDBB	100	63.3 (9.6) [b]	41.30%	94	68.3 (12.8)	43.70%	rs242557	Y	8	17192721
Fung 2006	Finland	Caucasian	UKPDBB	60	61.5 (8.8) [b]	40.80%	86	66.4 (9.2)	63.20%	rs242557	Y	8	17192721
Fung 2006	Taiwan	Asian	UKPDBB	56	61.7 (10.9) [b]	47.10%	114	59.0 (10.1)	43.80%	rs242557	Y	8	17192721
Vandrovcova 2009	UK	Caucasian	Autopsy confirmed	324	-	-	180	-	-	rs242557	Y	7	18162161
Vandrovcova 2009	UK	Caucasian	-	248	-	-	480	-	-	rs242557	Y	7	18162161
Das 2009	India	Asian	UKPDBB	301	45 (11) [b]	25.00%	243	49 (8)	21.00%	rs242557	Y	8	19450659
Satake 2009	Japan	Asian	-	1078	58.8 (10.1) [d]	54.92%	2628	49.9 (14.2)	45.24%	rs242557	Y	9	19915576
Satake 2009	Japan	Asian	-	612	43.0 (13.8) [b]	50.00%	14139	60.1 (12.6)	41.84%	rs242557	Y	9	19915576
Satake 2009	Japan	Asian	-	321	63.7 (9.7) [b]	55.45%	1614	59.1 (19.1)	55.27%	rs242557	Y	9	19915576
Wider 2010	Ireland	Caucasian	Gelb et al., 1999	360	51.8 (10.5) [b]	40.00%	437	65.0 (24.3)	64.00%	rs242557	Y	8	19912324
Wider 2010	US	Caucasian	Gelb et al., 1999	378	62.0 (12.2) [b]	43.00%	409	72.2 (10.8)	47.00%	rs242557	Y	8	19912324
Wider 2010	Norway	Caucasian	Gelb et al., 1999	480	58.9 (11.0) [b]	39.00%	555	70.6 (12.5)	44.00%	rs242557	Y	8	19912324
Ezquerra 2011	Spain	Caucasian	Hughes et al., 1992	505	56.6 (10.9) [b]	46.00%	233	68.8 (9)	48.00%	rs242557	-	8	19879020
Elbaz 2011	Australia	Caucasian	Bower et al., 1999	926	59.4 (11.4) [b]	38.00%	711	66.6 (9.9)	64.00%	rs242557	Y	8	21391235
Elbaz 2011	France	Caucasian	Gelb et al., 1999	558	54.7 (11.5) [b]	46.00%	141	65.2 (11.0)	55.00%	rs242557	Y	8	21391235
Elbaz 2011	Germany	Caucasian	UKPDBB	1084	-	56.84%	673	-	52.15%	rs242557	Y	8	21391235
Elbaz 2011	Greece	Caucasian	Bower et al., 1999, Gelb et al., 1999	456	-	52.74%	373	-	48.78%	rs242557	Y	8	21391235
Elbaz 2011	Italy	Caucasian	UKPDBB, Gelb et al., 1999	470	-	52.07%	361	-	50.14%	rs242557	Y	8	21391235
Elbaz 2011	Poland	Caucasian	UKPDBB	347	57.1 (11.6) [b]	38.00%	338	64.3 (15.7)	54.00%	rs242557	Y	8	21391235
Elbaz 2011	Sweden	Caucasian	Gelb et al., 1999	159	65.7 (11.0) [b]	44.00%	180	73.7 (10.1)	56.00%	rs242557	Y	8	21391235
Elbaz 2011	US	Caucasian	UKPDBB	376	62.1 (11.9) [b]	45.00%	364	72.9 (10.8)	48.00%	rs242557	Y	8	21391235
Seto-Salvia 2011	Spain	Caucasian	Hughes et al., 1992	202	58.1 (10.8) [b]	46.10%	374	81.3 (6.9)	68.20%	rs242557	Y	7	21403021
Chen 2015	Chinese	Asian	UKPDBB	1261	-	44.49%	830	-	-	rs242557	Y	7	26303052
Fung 2006	Greece	Caucasian	UKPDBB	100	63.3 (9.6) [b]	41.30%	94	68.3 (12.8)	43.70%	rs3785883	Y	8	17192721
Fung 2006	Finland	Caucasian	UKPDBB	60	61.5 (8.8) [b]	40.80%	86	66.4 (9.2)	63.20%	rs3785883	Y	8	17192721
Fung 2006	Taiwan	Asian	UKPDBB	56	61.7 (10.9) [b]	47.10%	114	59.0 (10.1)	43.80%	rs3785883	Y	8	17192721
Vandrovcova 2009	UK	Caucasian	Autopsy confirmed	324	-	-	180	-	-	rs3785883	Y	7	18162161

Vandrovcova 2009	UK	Caucasian	-	248	-	-	480	-	-	rs3785883	Y	7	18162161
Seto-Salvia 2011	Spain	Caucasian	Hughes et al., 1992	202	58.1 (10.8) [b]	46.10%	374	81.3 (6.9)	68.20%	rs3785883	Y	7	21403021
Fung 2006	Greece	Caucasian	UKPDBB	100	63.3 (9.6) [b]	41.30%	94	68.3 (12.8)	43.70%	rs2471738	Y	8	17192721
Fung 2006	Finland	Caucasian	UKPDBB	60	61.5 (8.8) [b]	40.80%	86	66.4 (9.2)	63.20%	rs2471738	Y	8	17192721
Fung 2006	Taiwan	Asian	UKPDBB	56	61.7 (10.9) [b]	47.10%	114	59.0 (10.1)	43.80%	rs2471738	Y	8	17192721
Vandrovcova 2009	UK	Caucasian	Autopsy confirmed	324	-	-	180	-	-	rs2471738	Y	7	18162161
Vandrovcova 2009	UK	Caucasian	-	248	-	-	480	-	-	rs2471738	Y	7	18162161
Das 2009	India	Asian	UKPDBB	301	45 (11) [b]	25.00%	243	49 (8)	21.00%	rs2471738	Y	8	19450659
Ezquerra 2011	Spain	Caucasian	Hughes et al., 1992	505	56.6 (10.9) [b]	46.00%	233	68.8 (9)	48.00%	rs2471738	-	8	19879020
Seto-Salvia 2011	Spain	Caucasian	Hughes et al., 1992	202	58.1 (10.8) [b]	46.10%	374	81.3 (6.9)	68.20%	rs2471738	Y	7	21403021
Fung 2006	Greece	Caucasian	UKPDBB	100	63.3 (9.6) [b]	41.30%	94	68.3 (12.8)	43.70%	rs7521	Y	8	17192721
Fung 2006	Finland	Caucasian	UKPDBB	60	61.5 (8.8) [b]	40.80%	86	66.4 (9.2)	63.20%	rs7521	Y	8	17192721
Fung 2006	Taiwan	Asian	UKPDBB	56	61.7 (10.9) [b]	47.10%	114	59.0 (10.1)	43.80%	rs7521	Y	8	17192721
Vandrovcova 2009	UK	Caucasian	Autopsy confirmed	324	-	-	180	-	-	rs7521	Y	7	18162161
Vandrovcova 2009	UK	Caucasian	-	248	-	-	480	-	-	rs7521	Y	7	18162161
Das 2009	India	Asian	UKPDBB	301	45 (11) [b]	25.00%	243	49 (8)	21.00%	rs7521	Y	8	19450659
Ezquerra 2011	Spain	Caucasian	Hughes et al., 1992	505	56.6 (10.9) [b]	46.00%	233	68.8 (9)	48.00%	rs7521	-	8	19879020
Seto-Salvia 2011	Spain	Caucasian	Hughes et al., 1992	202	58.1 (10.8) [b]	46.10%	374	81.3 (6.9)	68.20%	rs7521	Y	7	21403021
Maraganore 2001	US	Caucasian	UKPDBB	308	64(31–91) [b]	38.24%	190	72	62.24%	H2	-	7	11706972
Farrer 2002	Norway	Caucasian	Gelb et al., 1999	96	60(25–80) [b]	37.50%	68	80	54.41%	H2	Y	7	11958849
Clark 2003	US (mixed)	Caucasian	UKPDBB	60	75.1 (8.9) [c]	51.70%	458	75.7 (6.0)	64.20%	H2	Y	8	12865131
Clark 2003	US (mixed)	Caucasian	UKPDBB	84	67.8 (10.3) [c]	56.00%	458	75.7 (6.0)	64.20%	H2	Y	8	12865131
Peplonska 2003	Poland	Caucasian	UKPDBB	100	64.7 (8.5) [b]	38.00%	100	71.2 (5.9)	79.00%	H2	-	8	12932819
Levecque 2004	France	Caucasian	de Rijk et al., 1996	208	63 (7) [b]	43.27%	483	67 (7)	41.20%	H2	Y	8	14966169
Kwok 2004	Australia	Caucasian	Calne et al., 1992	206	62 [b]	-	169	-	-	H2	-	7	14991810
Skipper 2004	Norway	Caucasian	Gelb et al., 1999	296	59 (8) [b]	-	441	80 (6)	-	H2	-	7	15297935
Johansson 2005	Sweden	Caucasian	UKPDBB	105	59 (10.9) [b]	42.90%	160	69 (9.2)	60.60%	H2	Y	7	16909000
Fidani 2006	Greek	Caucasian	UKPDBB	133	52.3 [b]	36.84%	113	72.4	49.10%	H2	Y	7	16552760
Fung 2006	Greece	Caucasian	UKPDBB	100	63.3 (9.6) [b]	41.30%	94	68.3 (12.8)	43.70%	H2	Y	8	17192721
Fung 2006	Finland	Caucasian	UKPDBB	60	61.5 (8.8) [b]	40.80%	86	66.4 (9.2)	63.20%	H2	Y	8	17192721
Winkler 2007	Serbia	Caucasian	UKPDBB	191	47.7 (9.7) [b]	39.30%	156	45.6 (15.7)	55.80%	H2	Y	8	17637803

Winkler 2007	Germany	Caucasian	UKPDBB	256	46.4 (12.2) [b]	41.60%	162	50.2 (11.8)	47.50%	H2	Y	8	17637803
Camuzat 2008	Guadeloupe	African	-	40	70.2 (12.4) [c]	47.50%	132	65.01 (11)	58.47%	H2	Y	7	18785640
Vandrovcova 2009	UK	Caucasian	Autopsy confirmed	324	-	-	180	-	-	H2	Y	7	18162161
Vandrovcova 2009	UK	Caucasian	-	248	-	-	480	-	-	H2	Y	7	18162161
Das 2009	India	Asian	UKPDBB	301	45 (11) [b]	25.00%	243	49 (8)	21.00%	H2	Y	8	19450659
Refenes 2009	Greek	Caucasian	UKPDBB	122	64.5 (10.7) [c]	41.00%	123	63.7 (17.2)	33.30%	H2	Y	7	19558713
Simon-Sanchez 2009	US,UK , Germany	Caucasian	UKPDBB	5074	-	35.94%	8551	-	44.04%	H2	Y	8	19915575
Wider 2010	Ireland	Caucasian	Gelb et al., 1999	346	51.8 (10.5) [b]	40.00%	416	65.0 (24.3)	64.00%	H2	Y	8	19912324
Wider 2010	US	Caucasian	Gelb et al., 1999	361	62.0 (12.2) [b]	43.00%	405	72.2 (10.8)	47.00%	H2	Y	8	19912324
Wider 2010	Norway	Caucasian	Gelb et al., 1999	475	58.9 (11.0) [b]	39.00%	546	70.6 (12.5)	44.00%	H2	Y	8	19912324
Edwards 2010	US	Caucasian	UKPDBB	1752	-	48.57%	1745	-	50.95%	H2	Y	8	20070850
Kalinderi 2011	Greek	Caucasian	UKPDBB	196	52.9 [b]	35.71%	163	71.6	58.90%	H2	Y	7	19573950
Ezquerra 2011	Spain	Caucasian	Hughes et al., 1992	505	56.6 (10.9) [b]	46.00%	233	68.8 (9)	48.00%	H2	-	8	19879020
Consortium 2011	UK	Caucasian	UKPDBB	1705	65.8 [b]	27.04%	5175	-	-	H2	Y	7	21044948
Consortium 2011	France	Caucasian	UKPDBB	1039	-	-	1984	-	-	H2	Y	7	21044948
Simon-Sanchez 2011	Netherlands	Caucasian	-	772	55.3 (16–84) [c]	36.40%	2024	53.75(45-95)	56.18%	H2	-	7	21248740
Elbaz 2011	Australia	Caucasian	Bower et al., 1999	929	59.4 (11.4) [b]	38.00%	713	66.6 (9.9)	64.00%	H2	Y	8	21391235
Elbaz 2011	France	Caucasian	Gelb et al., 1999	563	54.7 (11.5) [b]	46.00%	143	65.2 (11.0)	55.00%	H2	Y	8	21391235
Elbaz 2011	Germany	Caucasian	UKPDBB	1089	-	56.84%	675	-	52.15%	H2	Y	8	21391235
Elbaz 2011	Greece	Caucasian	Bower et al., 1999, Gelb et al., 1999	457	-	52.74%	410	-	48.78%	H2	Y	8	21391235
Elbaz 2011	Italy	Caucasian	UKPDBB, Gelb et al., 1999	482	-	52.07%	365	-	50.14%	H2	Y	8	21391235
Elbaz 2011	Poland	Caucasian	UKPDBB	349	57.1 (11.6) [b]	38.00%	340	64.3 (15.7)	54.00%	H2	Y	8	21391235
Elbaz 2011	Sweden	Caucasian	Gelb et al., 1999	164	65.7 (11.0) [b]	44.00%	180	73.7 (10.1)	56.00%	H2	Y	8	21391235
Elbaz 2011	US	Caucasian	UKPDBB	378	62.1 (11.9) [b]	45.00%	364	72.9 (10.8)	48.00%	H2	Y	8	21391235
Seto-Salvia 2011	Spain	Caucasian	Hughes et al., 1992	202	58.1 (10.8) [b]	46.10%	374	81.3 (6.9)	68.20%	H2	Y	7	21403021
Mata 2011	Spain	Caucasian	UKPDBB	1445	60.0 (12.2) [b]	44.20%	1161	68.9 (11.2)	58.40%	H2	Y	8	21425343
Trotta 2012	Italy	Caucasian	UKPDBB	904	56.1 (11.0) [b]	39.90%	891	62.4 (14.6)	65.50%	H2	Y	7	22104010
Emelyanov 2013	Russia	Caucasian	-	244	64.1 (9.8) [d]	54.51%	308	67.7 (8.8)	56.17%	H2	Y	7	23830801

Sonmez 2015	Turkey	Caucasian	-	583	65.48 (11.109) [d]	40.48%	369	65.43 (13.151)	39.90%	H2	Y	8	25168738
Cervera-Carles 2016	Spain	Caucasian	Hughes et al., 1992	330	69.4 (9.2) [b]	44.60%	325	69.4 (9.2)	44.60%	H2	-	7	26453547
Fung 2006	Greece	Caucasian	UKPDBB	100	63.3 (9.6) [b]	41.30%	94	68.3 (12.8)	43.70%	H1c	Y	8	17192721
Fung 2006	Finland	Caucasian	UKPDBB	60	61.5 (8.8) [b]	40.80%	86	66.4 (9.20	63.20%	H1c	Y	8	17192721
Zabetian 2007	US	Caucasian	UKPDBB	1762	58.7 (11.6) [b]	32.30%	2010	67.4 (18.3)	62.70%	H1c	Y	8	17514749
Vandrovcova 2009	UK	Caucasian	-	572	-	-	660	-	-	H1c	Y	7	18162161
Seto-Salvia 2011	Spain	Caucasian	Hughes et al., 1992	202	58.1 (10.8) [b]	46.10%	374	81.3 (6.9)	68.20%	H1c	Y	7	21403021

Abbreviation: N, the number of cases/controls; SD, standard deviation; NOS, Newcastle-Ottawa Scale; PMID, PubMed-Indexed for MEDLINE; US, the United States; UK, the United Kingdom; UKPDBB, the UK Parkinson's Disease Society Brain Bank clinical diagnostic criteria; - , not obtained.

[a] Mean age at death (range)

[b] Mean age at onset

[c] Mean age at examination

[d] Not indicated

Supplementary Table 4 Characteristics of included studies in PSP

Author/ Year	State	Ethnicity	Diagnostic criteria	Cases			Controls			SNP/ Haplotype	HWE (Y/N)	NOS	PMID
				N	Mean age (SD/range)	Female %	N	Mean age (SD/range)	Female %				
Pittman 2005	US	Caucasian	Autopsy confirmed	238	75.3 [d]	50.00%	131	79.9	50.00%	rs1467967	Y	8	15792962
Pittman 2005	UK	Caucasian	Autopsy confirmed	83	73.5 [d]	37.00%	169	76	49.00%	rs1467967	Y	8	15792962
Cruchaga 2009	Spain	Caucasian	Tolosa et al., 1994, autopsy confirmed	127	-	-	190	70.8 (7.2)	52.10%	rs1467967	Y	8	19022385
Pittman 2005	US	Caucasian	Autopsy confirmed	238	75.3 [d]	50.00%	131	79.9	50.00%	rs242557	Y	8	15792962
Pittman 2005	UK	Caucasian	Autopsy confirmed	83	73.5 [d]	37.00%	169	76	49.00%	rs242557	Y	8	15792962
Cruchaga 2009	Spain	Caucasian	Tolosa et al., 1994, autopsy confirmed	127	-	-	190	70.8 (7.2)	52.10%	rs242557	Y	8	19022385
Hoglinger 2011	Europe	Caucasian	Autopsy confirmed	1069	-	45.00%	2964	-	47.70%	rs242557	Y	8	21685912
Hoglinger 2011	Europe	Caucasian	Litvan et al., 1996, autopsy confirmed	1051	-	47.00%	3560	-	47.00%	rs242557	Y	8	21685912
Pittman 2005	US	Caucasian	Autopsy confirmed	238	75.3 [d]	50.00%	131	79.9	50.00%	rs3785883	Y	8	15792962
Pittman 2005	UK	Caucasian	Autopsy confirmed	83	73.5 [d]	37.00%	169	76	49.00%	rs3785883	Y	8	15792962
Pittman 2005	US	Caucasian	Autopsy confirmed	238	75.3 [d]	50.00%	131	79.9	50.00%	rs2471738	Y	8	15792962

Pittman 2005	UK	Caucasian	Autopsy confirmed	83	73.5 [d]	37.00%	169	76	49.00%	rs2471738	Y	8	15792962
Cruchaga 2009	Spain	Caucasian	Tolosa et al., 1994, autopsy confirmed	127	-	-	190	70.8 (7.2)	52.10%	rs2471738	Y	8	19022385
Pittman 2005	US	Caucasian	Autopsy confirmed	238	75.3 [d]	50.00%	131	79.9	50.00%	rs7521	Y	8	15792962
Pittman 2005	UK	Caucasian	Autopsy confirmed	83	73.5 [d]	37.00%	169	76	49.00%	rs7521	Y	8	15792962
Cruchaga 2009	Spain	Caucasian	Tolosa et al., 1994, autopsy confirmed	127	-	-	190	70.8 (7.2)	52.10%	rs7521	Y	8	19022385
Baker 1999	-	Caucasian	Litvan et al., 1996, autopsy confirmed	64	65.3 [c]	-	145	63	-	H2	-	7	10072441
Ezquerra 1999	-	Caucasian	Tolosa et al., 1995	35	66.61 (5.8) [b]	45.71%	195	61.4 (13.9)	51.28%	H2	-	8	10580705
de Silva 2001	UK	Caucasian	Litvan et al., 1996, autopsy confirmed	42	-	-	70	72	-	H2	Y	7	11578815
de Silva 2003	Britain and western Europe	Caucasian	Litvan et al., 1996	49	-	-	62	-	-	H2	Y	6	12913211
Ezquerra 2004	Spain	Caucasian	Tolosa et al., 1995, Litvan et al., 1996	57	70 (5.5) [b]	54.39%	83	68.9 (7.5)	59.04%	H2	-	8	14707330
Pittman 2005	US	Caucasian	Autopsy confirmed	238	75.3 [d]	50.00%	131	79.9	50.00%	H2	Y	8	15792962
Pittman 2005	UK	Caucasian	Autopsy confirmed	83	73.5 [d]	37.00%	169	76	49.00%	H2	Y	8	15792962
Rademakers 2005	US	Caucasian	Autopsy confirmed	274	-	-	424	-	-	H2	Y	7	16195395
Ezquerra 2007	Spain	Caucasian	Autopsy confirmed	13	66.6 (5.5) [b]	38.46%	6	-	66.67%	H2	-	5	17320831
Webb 2008	US	Caucasian	Litvan et al., 1996	36	-	44.44%	98	73	57.14%	H2	Y	7	19001166
Cruchaga 2009	Spain	Caucasian	Tolosa et al., 1994, autopsy confirmed	127	-	-	190	70.8 (7.2)	52.10%	H2	Y	8	19022385
Hoglinger 2011	Europe	Caucasian	Autopsy confirmed	1069	-	45.00%	2964	-	47.70%	H2	Y	8	21685912
Hoglinger 2011	Europe	Caucasian	Litvan et al., 1996, autopsy confirmed	1051	-	47.00%	3560	-	47.00%	H2	Y	8	21685912
Cervera-Carles 2016	Spain	Caucasian	Litvan et al., 1996	96	66.8 (9) [b]	54.20%	325	69.4 (9.2)	44.60%	H2	-	7	26453547
Pittman 2005	US	Caucasian	Autopsy confirmed	238	75.3 [d]	50.00%	131	79.9	50.00%	H1c	Y	8	15792962
Pittman 2005	UK	Caucasian	Autopsy confirmed	83	73.5 [d]	37.00%	169	76	49.00%	H1c	Y	8	15792962
Cruchaga 2009	Spain	Caucasian	Tolosa et al., 1994, autopsy confirmed	127	-	-	190	70.8 (7.2)	52.10%	H1c	Y	8	19022385

Abbreviation: N, the number of cases/controls; SD, standard deviation; NOS, Newcastle-Ottawa Scale; PMID, PubMed-Indexed for MEDLINE; US, the United States; UK, the United Kingdom; - , not obtained.

[a] Mean age at death (range)

[b] Mean age at onset

[c] Mean age at examination

[d] Not indicated

Supplementary Table 5 Characteristics of included studies in CBD

Author/ Year	State	Ethnicity	Diagnostic criteria	Cases			Controls			SNP/ Haplotype	HWE (Y/N)	NOS	PMID
				N	Mean age (SD/range)	Female %	N	Mean age (SD/range)	Female %				
Pittman 2005	US	Caucasian	Autopsy confirmed	44	71.3 [d]	50.00%	131	79.9	50.00%	rs1467967	Y	8	15792962
Cruchaga 2009	Spain	Caucasian	Watts et al, 1997	16	64.9 (9.2) [b]	32.00%	190	70.8 (7.2)	52.10%	rs1467967	Y	8	19022385
Pittman 2005	US	Caucasian	Autopsy confirmed	44	71.3 [d]	50.00%	131	79.9	50.00%	rs242557	Y	8	15792962
Cruchaga 2009	Spain	Caucasian	Watts et al, 1997	16	64.9 (9.2) [b]	32.00%	190	70.8 (7.2)	52.10%	rs242557	Y	8	19022385
Pittman 2005	US	Caucasian	Autopsy confirmed	44	71.3 [d]	50.00%	131	79.9	50.00%	rs3785883	Y	8	15792962
Pittman 2005	US	Caucasian	Autopsy confirmed	44	71.3 [d]	50.00%	131	79.9	50.00%	rs2471738	Y	8	15792962
Cruchaga 2009	Spain	Caucasian	Watts et al, 1997	16	64.9 (9.2) [b]	32.00%	190	70.8 (7.2)	52.10%	rs2471738	Y	8	19022385
Pittman 2005	US	Caucasian	Autopsy confirmed	44	71.3 [d]	50.00%	131	79.9	50.00%	rs7521	Y	8	15792962
Cruchaga 2009	Spain	Caucasian	Watts et al, 1997	16	64.9 (9.2) [b]	32.00%	190	70.8 (7.2)	52.10%	rs7521	Y	8	19022385
Kouri 2014	US	Caucasian	Dickson et al, 2002	109	69.7 (8.7) [a]	47.70%	643	73.7 (11.8)	52.30%	rs7521	-	8	24121548
Houlden 2001	US	Caucasian	Autopsy confirmed	30	-	-	145	63	-	H2	Y	7	11425937
Houlden 2001	UK	Caucasian	Autopsy confirmed	13	-	-	75	68	-	H2	Y	7	11425937
Pittman 2005	US	Caucasian	Autopsy confirmed	44	71.3 [d]	50.00%	131	79.9	50.00%	H2	Y	8	15792962
Webb 2008	US	Caucasian	-	22	-	59.09%	98	73	57.14%	H2	Y	7	19001166
Cruchaga 2009	Spain	Caucasian	Watts et al, 1997	16	64.9 (9.2) [b]	32.00%	190	70.8 (7.2)	52.10%	H2	Y	8	19022385
Kouri 2014	US	Caucasian	Dickson et al, 2002	108	69.7 (8.7) [a]	47.70%	640	73.7 (11.8)	52.30%	H2	-	8	24121548
Cervera-Carles 2016	Spain	Caucasian	Watts et al, 1997	55	67.0 (10) [b]	52.70%	325	69.4 (9.2)	44.60%	H2	-	7	26453547
Pittman 2005	US	Caucasian	Autopsy confirmed	44	71.3 [d]	50.00%	131	79.9	50.00%	H1c	Y	8	15792962
Cruchaga 2009	Spain	Caucasian	Watts et al, 1997	16	64.9 (9.2) [b]	32.00%	190	70.8 (7.2)	52.10%	H1c	Y	8	19022385

Abbreviation: N, the number of cases/controls; SD, standard deviation; NOS, Newcastle-Ottawa Scale; PMID, PubMed-Indexed for MEDLINE; US, the United States; UK, the United Kingdom; - , not obtained.

[a] Mean age at death (range)

[b] Mean age at onset

[c] Mean age at examination

[d] Not indicated

Supplementary Table 6 Characteristics of included studies in FTD

Author/ Year	State	Ethnicity	Diagnostic criteria	Cases			Controls			Haplotype	HWE	NOS	PMID
				N	Mean age (SD/range)	Female %	N	Mean age (SD/range)	Female %		(Y/N)		
Verpillat 2002	France	Caucasian	Lund-Manchester	100	60.6 (9.3) [b]	56	79	60.0 (8.8)	70	H2	Y	8	12056929
Verpillat 2002	France	Caucasian	Lund-Manchester	91	59.7 (9.3) [b]	58.00%	402	66.6 (10.2)	52.00%	H2	Y	8	12447938
Hughes 2003	UK	Caucasian	Lund-Manchester	58	56.8 (10.9) [b]	-	168	-	-	H2	-	7	12710929
Sobrido 2003	US	Caucasian	Miller et al, 1997	45	54.2(25-75) [b]	47.92%	36	-	-	H2	-	8	12756133
Ghidoni 2006	Italy	Caucasian	Lund-Manchester	53	66.8 (10.4) [c]	60.40%	99	69.2 (6.5)	66.60%	H2	Y	8	16410051
Ghidoni 2006	Italy	Caucasian	Lund-Manchester	50	66.9 (9.5) [c]	58.00%	99	69.2 (6.5)	66.60%	H2	Y	8	16410051
Johansson 2005	Sweden	Caucasian	Lund-Manchester	96	61 (8.3) [b]	60.40%	186	72 (9.2)	56.50%	H2	Y	7	16909000
Webb 2008	US	Caucasian	Neary et al, 1998	56	-	42.86%	98	73	57.14%	H2	Y	7	19001166
Kaivorinne 2008	Finland.	Caucasian	Lund-Manchester	59	58.5 [d]	51.00%	198	40.6	-	H2	-	7	19091059

Abbreviation: N, the number of cases/controls; SD, standard deviation; NOS, Newcastle-Ottawa Scale; PMID, PubMed-Indexed for MEDLINE; US, the United States; UK, the United Kingdom; Lund-Manchester, the Lund and Manchester groups research criteria for frontotemporal dementia; - , not obtained.

[a] Mean age at death (range)

[b] Mean age at onset

[c] Mean age at examination

[d] Not indicated

Supplementary Table 7 Characteristics of included studies in ALS

Author/ Year	State	Ethnicity	Diagnostic criteria	Cases			Controls			Haplotype	HWE	NOS	PMID
				N	Mean age (SD/range)	Female %	N	Mean age (SD/range)	Female %		(Y/N)		
Hughes 2003	UK	Caucasian	El Escarol	108	-	-	168	-	-	H2	-	7	12710929
Taes 2010	Belgium	Caucasian	El Escarol	342	58.6 (12.6) [b]	40.00%	1344	53.4 (21.1)	47.00%	H2	Y	8	20498436
Taes 2010	Netherlands	Caucasian	El Escarol	1058	60.4 (12.6) [b]	40.00%	1066	63.3 (9.4)	42.00%	H2	Y	8	20498436
Taes 2010	Italy	Caucasian	El Escarol	266	60.5 (11.0) [b]	46.00%	1189	66.8 (14.9)	54.00%	H2	Y	8	20498436
Taes 2010	Poland	Caucasian	El Escarol	200	53.4 (13.1) [b]	49.00%	537	57.2 (17.6)	54.00%	H2	Y	8	20498436
Taes 2010	Sweden	Caucasian	El Escarol	476	61.8 (12.7) [b]	42.00%	486	59.8 (14)	46.00%	H2	Y	8	20498436

Taes 2010	US	Caucasian	El Escarol	949	53.9 (12.9) [b]	37.00%	937	61.1 (13.9)	48.00%	H2	Y	8	20498436
Taes 2010	UK	Caucasian	El Escarol	237	56.8 (12.5) [b]	39.00%	3131	55.6 (12.2)	37.00%	H2	Y	8	20498436

Abbreviation: N, the number of cases/controls; SD, standard deviation; NOS, Newcastle-Ottawa Scale; PMID, PubMed-Indexed for MEDLINE; US, the United States; UK, the United Kingdom; El Escarol, the criteria for the diagnosis of ALS; - , not obtained.

[a] Mean age at death (range)

[b] Mean age at onset

[c] Mean age at examination

[d] Not indicated

Supplementary Table 8 Meta-analysis of the *MAPT* polymorphisms on Alzheimer's disease

Groups	n [a]	OR(95%CI) [b]	I^2 [c]	P (Q) [c]	P(Egger) [d]
SNPs					
rs1467967	12	1.01(0.97-1.05)	32.1	0.1336	0.4891
rs242557	12	1.02(0.94-1.12)	65.2	0.0009	0.6323
rs3785883	11	0.89(0.77-1.02)	73.1	<0.0001	0.0917
rs2471738	11	1.04(1.00-1.09)	49.2	0.0326	0.4493
rs7521	11	1.00(0.97-1.03)	0	0.5235	0.7084
Haplotype/subhaplotype					
H2	39	0.94(0.91-0.97)	49.4	0.0003	0.4958
H1c	6	1.02(0.97-1.08)	45.5	0.1022	-

Abbreviations: OR, odds ratio; CI, confidence interval; SNP, single-nucleotide polymorphism; -, not obtained.

[a] Number of studies included

[b] The OR and 95 % CI are shown as calculated for the minor vs. major allele frequency using the Pearson χ^2 test or Fisher's exact test.

[c] I^2 and P value of Cochran Q for heterogeneity test

[d] P value of Egger's test for publication bias

Supplementary Table 9 Meta-analysis of the *MAPT* polymorphisms on Alzheimer's disease in Caucasian

Groups	n [a]	OR(95%CI) [b]	I^2 [c]	P (Q) [c]	P(Egger) [d]
SNPs					
rs1467967	11	1.01 (0.97-1.05)	35.5	0.1148	0.6272
rs242557	10	1.02(0.98-1.06)	46	0.0542	0.0654

rs3785883	10	0.87(0.76-1.00)	75.2	<0.0001	0.0499
rs2471738	10	1.08(0.98-1.20)	52.9	0.0244	0.3417
rs7521	10	1.00(0.97-1.03)	0	0.4895	0.9317
Haplotype/subhaplotype					
H2	38	0.96(0.90-1.02)	50.5	0.0002	0.5797
H1c	6	1.02(0.97-1.08)	45.5	0.1022	-

Abbreviations: OR, odds ratio; CI, confidence interval; SNP, single-nucleotide polymorphism; -, not obtained.

[a] Number of studies included

[b] The OR and 95 % CI are shown as calculated for the minor vs. major allele frequency using the Pearson χ^2 test or Fisher's exact test.

[c] I^2 and P value of Cochran Q for heterogeneity test

[d] P value of Egger's test for publication bias

Supplementary Table 10 Meta-analysis of the *MAPT* polymorphisms on Alzheimer's disease in Asian

Groups	n [a]	OR(95%CI) [b]	I^2 [c]	P (Q) [c]	P(Egger) [d]
SNPs					
rs1467967	1	0.85(0.58-1.26)	-	-	-
rs242557	2	0.71(0.47-1.08)	76.4	0.0397	-
rs3785883	1	1.28(0.76-2.16)	-	-	-
rs2471738	1	0.88(0.57-1.37)	-	-	-
rs7521	1	0.77(0.41-1.45)	-	-	-
Haplotype/subhaplotype					
H2	1	2.36(0.10-58.12)	-	-	-

Abbreviations: OR, odds ratio; CI, confidence interval; SNP, single-nucleotide polymorphism; -, not obtained.

[a] Number of studies included

[b] The OR and 95 % CI are shown as calculated for the minor vs. major allele frequency using the Pearson χ^2 test or Fisher's exact test.

[c] I^2 and P value of Cochran Q for heterogeneity test

[d] P value of Egger's test for publication bias

Supplementary Table 11 Meta-analysis of the *MAPT* polymorphisms on Parkinson's disease

Groups	n [a]	OR(95%CI) [b]	I^2 [c]	P (Q) [c]	P(Egger) [d]
SNPs					

rs1467967	8	1.07(0.97-1.17)	2.1	0.4134	-
rs242557	23	1.02(0.98-1.06)	30.6	0.0826	0.1894
rs3785883	6	1.03(0.89-1.18)	38.1	0.152	-
rs2471738	8	0.93(0.83-1.04)	29.1	0.1958	-
rs7521	8	1.06(0.96-1.16)	28.5	0.2005	-
Haplotype/subhaplotype					
H2	43	0.76(0.74-0.79)	42.5	0.0021	0.7561
H1c	5	1.07(0.97-1.19)	15.4	0.3163	-

Abbreviations: OR, odds ratio; CI, confidence interval; SNP, single-nucleotide polymorphism; -, not obtained.

[a] Number of studies included

[b] The OR and 95 % CI are shown as calculated for the minor vs. major allele frequency using the Pearson χ^2 test or Fisher's exact test.

[c] I^2 and P value of Q test for heterogeneity test

[d] P value of Egger's test for publication bias

Supplementary Table 12 Meta-analysis of the *MAPT* polymorphisms on Parkinson's disease in Caucasian

Groups	n [a]	OR(95%CI) [b]	I^2 [c]	P (Q) [c]	P(Egger) [d]
SNPs					
rs1467967	6	1.05(0.95-1.18)	26.2	0.2376	-
rs242557	17	1.06(1.01-1.12)	3.5	0.4137	0.6017
rs3785883	5	1.01(0.87-1.17)	48.3	0.1018	-
rs2471738	6	0.94(0.83-1.07)	48.1	0.0866	-
rs7521	6	1.11(1.00-1.23)	0	0.4711	-
Haplotype/subhaplotype					
H2	41	0.76(0.74-0.79)	41	0.0039	0.3882
H1c	5	1.07(0.97-1.19)	15.4	0.3163	-

Abbreviations: OR, odds ratio; CI, confidence interval; SNP, single-nucleotide polymorphism; -, not obtained.

a Number of studies included

b The OR and 95 % CI are shown as calculated for the minor vs. major allele frequency using the Pearson χ^2 test or Fisher's exact test.

c I^2 and P value of Cochran Q for heterogeneity test

d P value of Egger's test for publication bias

Supplementary Table 13 Meta-analysis of the *MAPT* polymorphisms on Parkinson's disease in Asian

Groups	n [a]	OR(95%CI) [b]	I^2 [c]	P (Q) [c]	P(Egger) [d]
SNPs					
rs1467967	2	1.11(0.92-1.35)	0	0.7173	-
rs242557	6	0.97(0.91-1.02)	45.2	0.1041	-
rs3785883	1	1.16(0.76-1.77)	-	-	-
rs2471738	2	0.88(0.67-1.15)	0	0.8849	-
rs7521	2	0.84(0.67-1.05)	0	0.7749	-
Haplotype/subhaplotype					
H2	1	1.35(0.77-2.39)	-	-	-

Abbreviations: OR, odds ratio; CI, confidence interval; SNP, single-nucleotide polymorphism; -, not obtained.

a Number of studies included

b The OR and 95 % CI are shown as calculated for the minor vs. major allele frequency using the Pearson χ^2 test or Fisher's exact test.

c I^2 and P value of Cochran Q for heterogeneity test

d P value of Egger's test for publication bias

Supplementary Table 14 Meta-analysis of the *MAPT* polymorphisms on progressive supranuclear palsy

Groups	n [a]	OR(95%CI) [b]	I^2 [c]	P (Q) [c]	P(Egger) [d]
SNPs					
rs1467967	3	1.05(0.86-1.28)	32.2	0.2286	-
rs242557	5	1.96(1.71-2.25)	62.8	0.0294	-
rs3785883	2	0.95(0.52-1.72)	76.6	0.0387	-
rs2471738	3	1.85(1.48-2.31)	36.4	0.2077	-
rs7521	3	0.97(0.80-1.17)	32.9	0.2254	-
Haplotype/subhaplotype					
H2	14	0.20(0.18-0.23)	30.3	0.1343	0.2587
H1c	3	2.33(1.28-4.25)	79.3	0.008	-

Abbreviations: OR, odds ratio; CI, confidence interval; SNP, single-nucleotide polymorphism; -, not obtained.

[a] Number of studies included

[b] The OR and 95 % CI are shown as calculated for the minor vs. major allele frequency using the Pearson χ^2 test or Fisher's exact test.

[c] I^2 and P value of Q test for heterogeneity test

[d] P value of Egger's test for publication bias

Supplementary Table 15 Meta-analysis of the *MAPT* polymorphisms on corticobasal degeneration

Groups	n [a]	OR(95%CI) [b]	I^2 [c]	P (Q) [c]	P(Egger) [d]
SNPs					
rs1467967	2	1.18(0.78-1.79)	0	0.3995	-
rs242557	2	2.51(1.66-3.78)	0	0.4146	-
rs3785883	1	1.69(0.99-2.88)	-	-	-
rs2471738	2	2.07(1.32-3.23)	0	0.7024	-
rs7521	3	1.29(0.83-2.00)	60.4	0.0802	-
Haplotype/subhaplotype					
H2	7	0.30(0.23-0.41)	0	0.6908	-
H1c	2	2.57(1.51-4.40)	0	0.8457	-

Abbreviations: OR, odds ratio; CI, confidence interval; SNP, single-nucleotide polymorphism; -, not obtained.

[a] Number of studies included

[b] The OR and 95 % CI are shown as calculated for the minor vs. major allele frequency using the Pearson χ^2 test or Fisher's exact test.

[c] I^2 and P value of Q test for heterogeneity test

[d] P value of Egger's test for publication bias

Supplementary Table 16 Meta-analysis of the H2 haplotype on frontotemporal dementia

Haplotypes	n [a]	OR(95%CI) [b]	I^2 [c]	P (Q) [c]	P(Egger) [d]
H2	9	1.02(0.78-1.32)	55.5	0.0215	-

Abbreviations: OR, odds ratio; CI, confidence interval; -, not obtained.

[a] Number of studies included

[b] The OR and 95 % CI are shown as calculated for the minor vs. major allele frequency using the Pearson χ^2 test or Fisher's exact test.

[c] I^2 and P value of Q test for heterogeneity test

[d] P value of Egger's test for publication bias

Supplementary Table 17 Meta-analysis of the H2 haplotype on amyotrophic lateral sclerosis

Haplotypes	n [a]	OR(95%CI) [b]	I^2 [c]	P (Q) [c]	P(Egger) [d]
H2	8	0.92(0.86-0.98)	0	0.452	-

Abbreviations: OR, odds ratio; CI, confidence interval; -, not obtained.

[a] Number of studies included

[b] The OR and 95 % CI are shown as calculated for the minor vs. major allele frequency using the Pearson χ^2 test or Fisher's exact test.

[c] I^2 and P value of Q test for heterogeneity test

[d] P value of Egger’s test for publication bias

Supplementary Figure 1 Meta-analysis of the *MAPT* polymorphisms on Alzheimer's disease

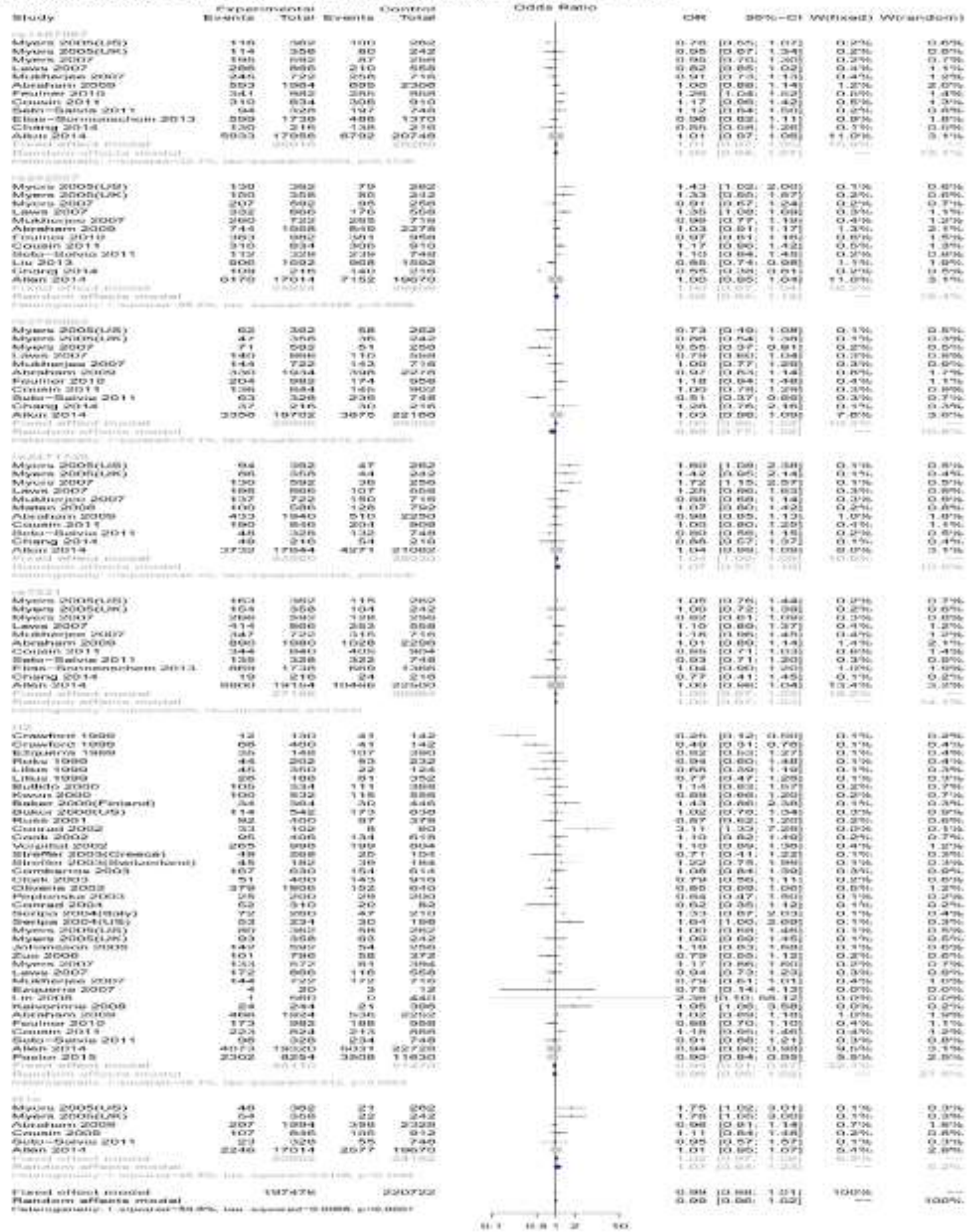

Supplementary Figure 2 Meta-analysis of the *MAPT* polymorphisms on Alzheimer's disease in Caucasian

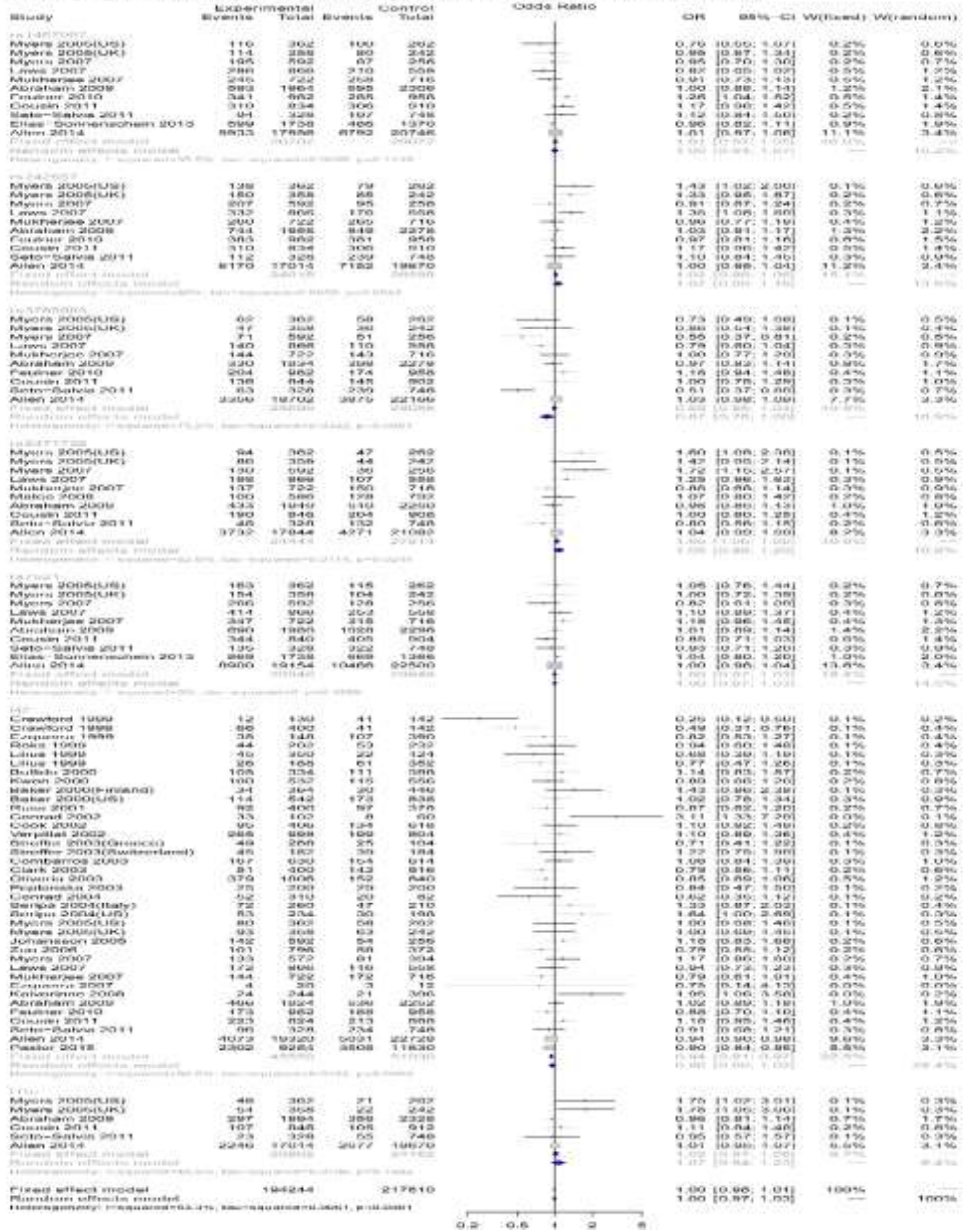

Supplementary Figure 3 Meta-analysis of the *MAPT* polymorphisms on Alzheimer's disease in Asian

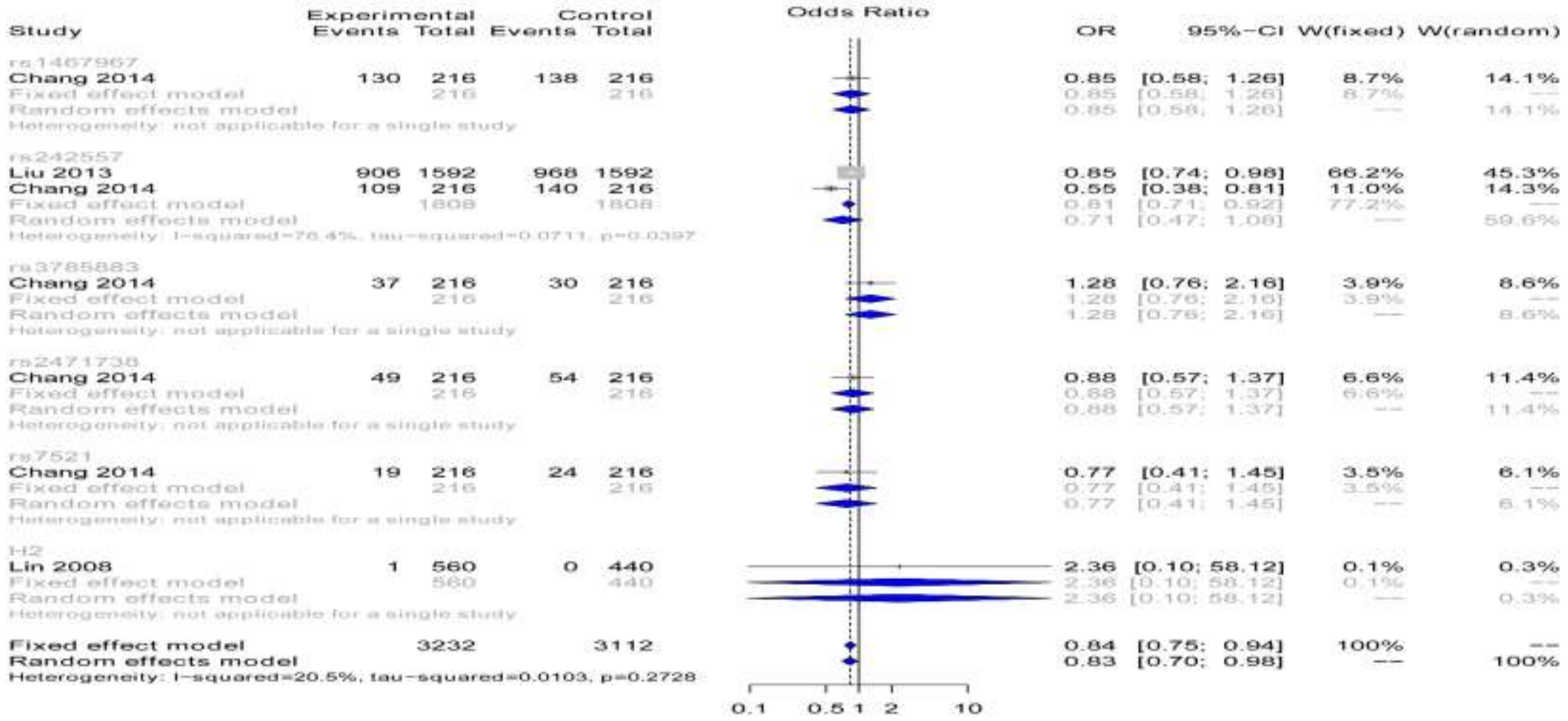

Supplementary Figure 4 Meta-analysis of the *MAPT* polymorphisms on Parkinson's disease

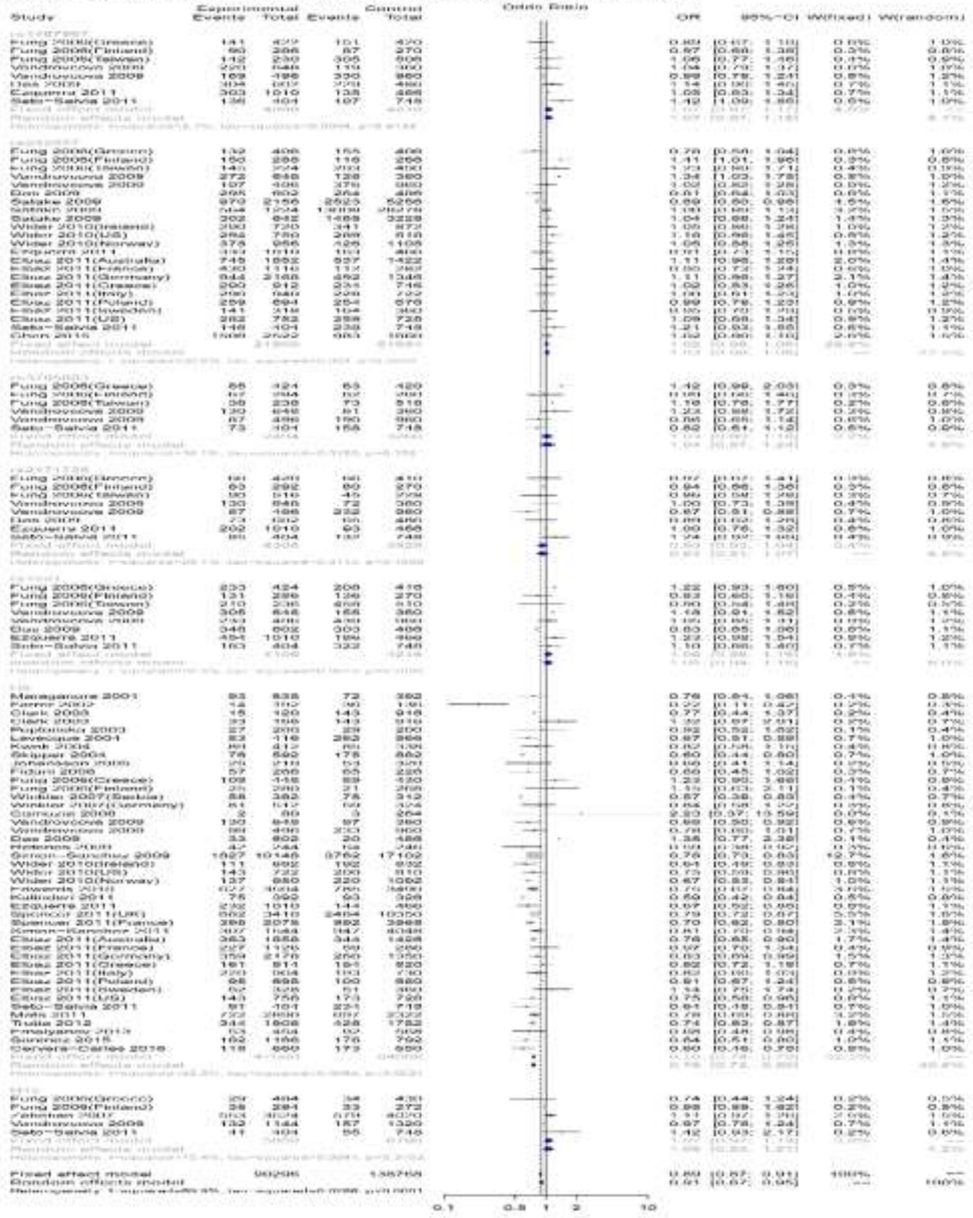

Supplementary Figure 5 Meta-analysis of the *MAPT* polymorphisms on Parkinson's disease in Caucasian

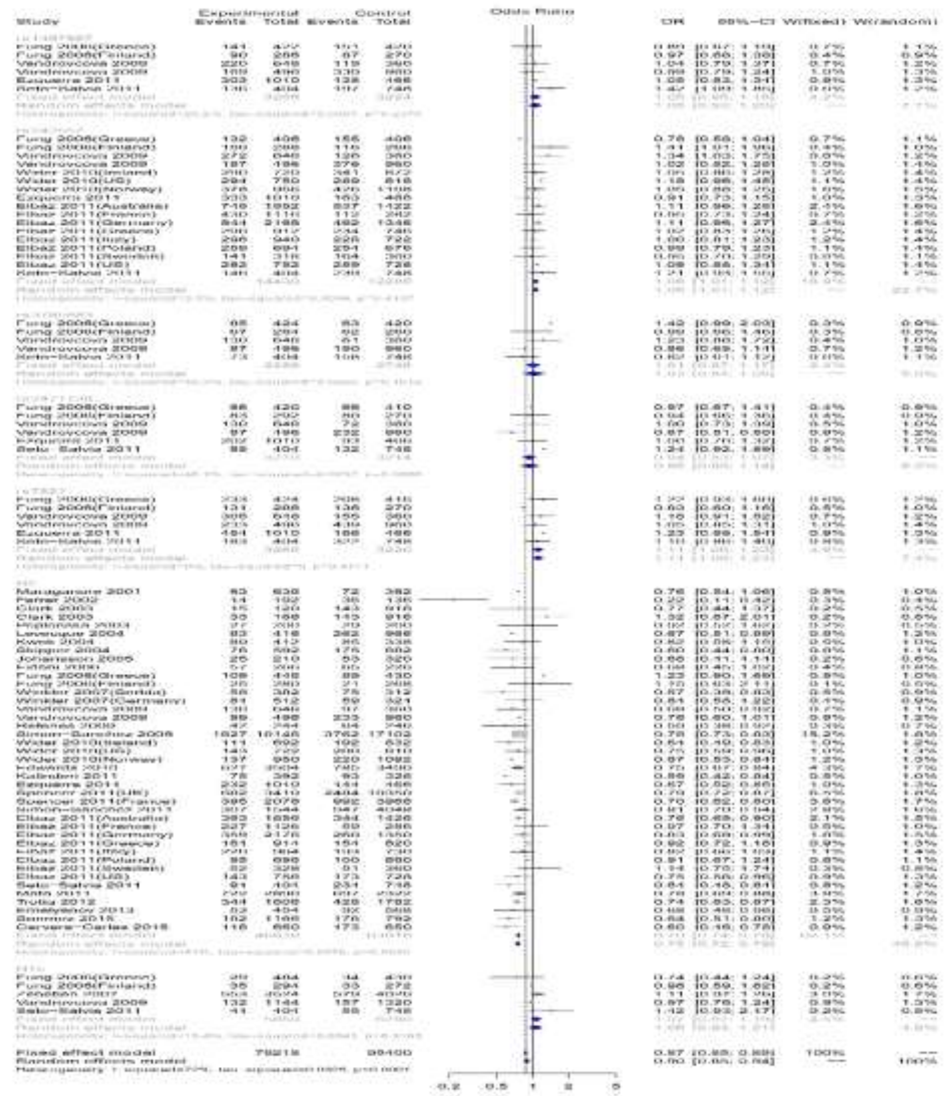

Supplementary Figure 6 Meta-analysis of the *MAPT* polymorphisms on Parkinson's disease in Asian

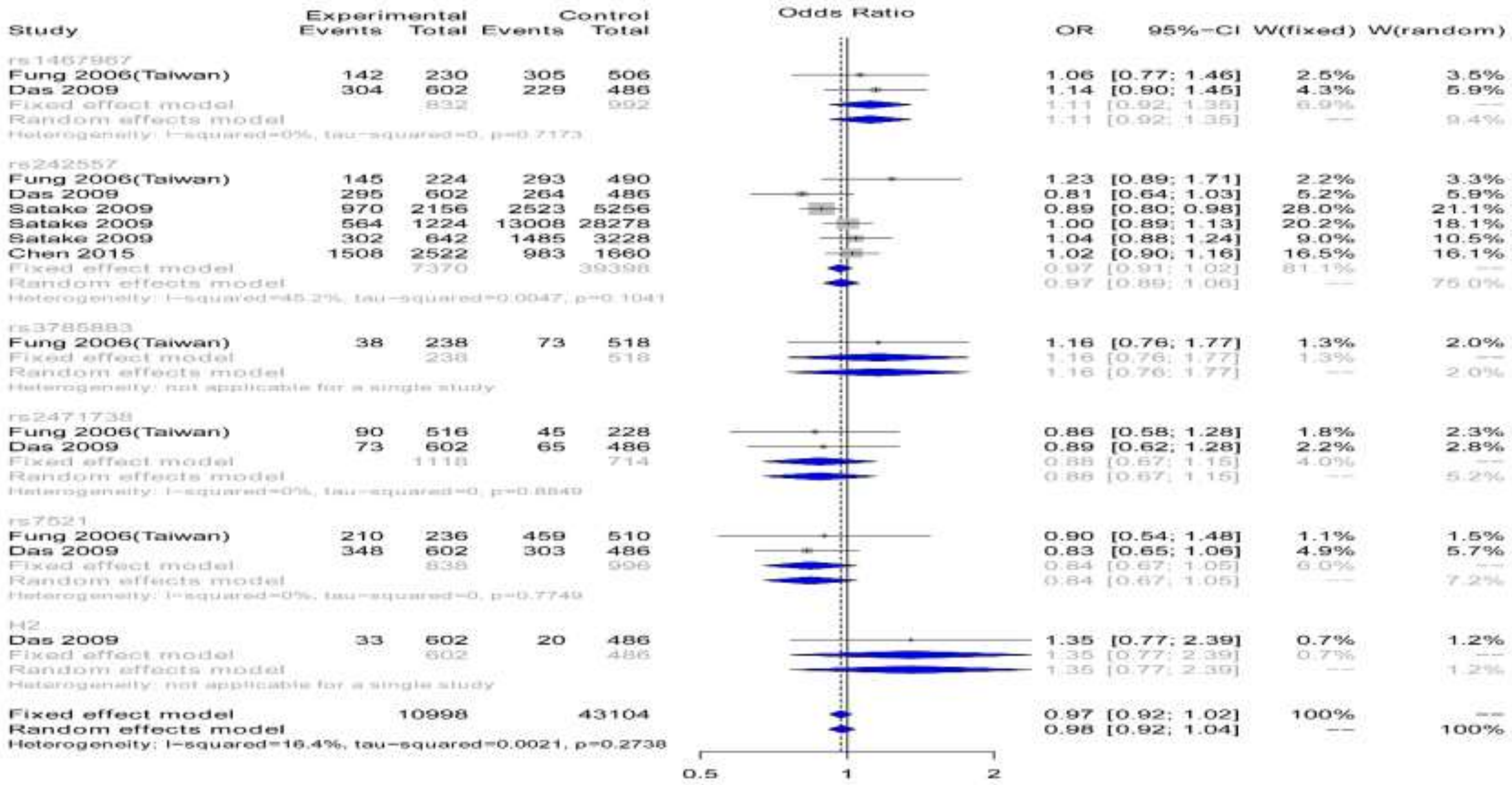

Supplementary Figure 7 Meta-analysis of the *MAPT* polymorphisms on progressive supranuclear palsy

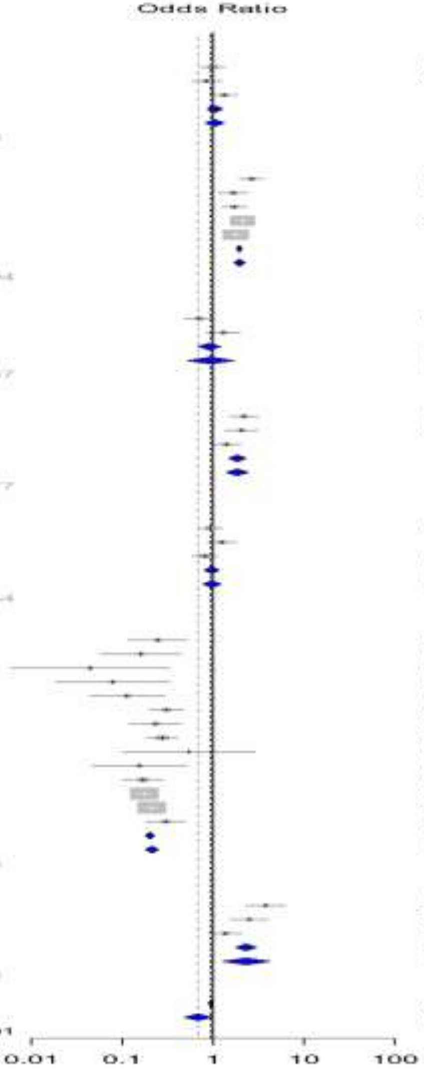

Study	Experimental Events	Experimental Total	Control Events	Control Total	OR	95%-CI	W(fixed)	W(random)
rs1467967								
Pittman 2005(US)	177	476	98	262	0.99	[0.73; 1.35]	2.0%	3.3%
Pittman 2005(UK)	53	166	120	338	0.85	[0.57; 1.26]	1.4%	3.2%
Cruchaga 2009	86	254	106	380	1.32	[0.94; 1.87]	1.4%	3.3%
Fixed effect model		896		980	1.05	[0.86; 1.28]	4.8%	--
Random effects model					1.05	[0.82; 1.34]	--	9.7%
Heterogeneity: I-squared=32.2%, tau-squared=0.015, p=0.2286								
rs242557								
Pittman 2005(US)	259	476	81	262	2.67	[1.94; 3.67]	1.2%	3.3%
Pittman 2005(UK)	80	166	121	338	1.67	[1.14; 2.43]	1.1%	3.2%
Cruchaga 2009	119	254	129	380	1.72	[1.24; 2.37]	1.4%	3.3%
Hoglinger 2011	1133	2138	2063	5928	2.11	[1.91; 2.34]	13.1%	3.4%
Hoglinger 2011	1040	2102	2528	7120	1.78	[1.61; 1.96]	14.9%	3.4%
Fixed effect model		5136		14028	1.94	[1.82; 2.08]	31.6%	--
Random effects model					1.96	[1.71; 2.25]	--	16.5%
Heterogeneity: I-squared=82.8%, tau-squared=0.0127, p=0.0294								
rs3785883								
Pittman 2005(US)	81	476	59	262	0.71	[0.48; 1.03]	1.6%	3.2%
Pittman 2005(UK)	42	166	70	338	1.30	[0.84; 2.01]	0.9%	3.2%
Fixed effect model		642		600	0.91	[0.69; 1.22]	2.5%	--
Random effects model					0.95	[0.52; 1.72]	--	6.4%
Heterogeneity: I-squared=76.6%, tau-squared=0.1419, p=0.0387								
rs2471738								
Pittman 2005(US)	157	476	48	262	2.19	[1.52; 3.17]	1.1%	3.2%
Pittman 2005(UK)	56	166	67	338	2.06	[1.36; 3.13]	0.7%	3.2%
Cruchaga 2009	66	254	76	380	1.40	[0.96; 2.05]	1.2%	3.2%
Fixed effect model		896		980	1.85	[1.48; 2.31]	3.0%	--
Random effects model					1.85	[1.40; 2.44]	--	9.7%
Heterogeneity: I-squared=36.4%, tau-squared=0.0222, p=0.2077								
rs7521								
Pittman 2005(US)	206	476	117	262	0.95	[0.70; 1.28]	2.2%	3.3%
Pittman 2005(UK)	85	166	154	338	1.25	[0.86; 1.82]	1.3%	3.2%
Cruchaga 2009	137	254	224	380	0.82	[0.59; 1.12]	2.1%	3.3%
Fixed effect model		896		980	0.97	[0.80; 1.17]	5.6%	--
Random effects model					0.97	[0.77; 1.23]	--	9.8%
Heterogeneity: I-squared=32.9%, tau-squared=0.0139, p=0.2254								
H2								
Baker 1999	8	128	62	290	0.25	[0.11; 0.53]	0.9%	2.9%
Ezquerra 1999	4	70	107	390	0.16	[0.06; 0.45]	0.8%	2.6%
de Silva 2001	1	84	30	140	0.04	[0.01; 0.33]	0.6%	1.6%
de Silva 2003	2	98	26	124	0.08	[0.02; 0.34]	0.6%	2.1%
Ezquerra 2004	5	114	48	166	0.11	[0.04; 0.29]	1.0%	2.7%
Pittman 2005(US)	40	476	60	262	0.31	[0.20; 0.48]	1.8%	3.2%
Pittman 2005(UK)	11	166	79	338	0.23	[0.12; 0.45]	1.2%	3.0%
Rademakers 2005	35	548	168	848	0.28	[0.19; 0.40]	3.2%	3.2%
Ezquerra 2007	4	26	3	12	0.55	[0.10; 2.94]	0.1%	1.9%
Webb 2008	3	72	43	196	0.15	[0.05; 0.52]	0.6%	2.4%
Cruchaga 2009	18	254	118	380	0.17	[0.10; 0.29]	2.2%	3.1%
Hoglinger 2011	109	2138	1393	5928	0.17	[0.14; 0.21]	17.9%	3.3%
Hoglinger 2011	126	2102	1652	7120	0.21	[0.17; 0.25]	18.1%	3.3%
Cervera-Carles 2016	19	192	173	650	0.30	[0.18; 0.50]	1.8%	3.1%
Fixed effect model		6468		16844	0.20	[0.18; 0.23]	50.6%	--
Random effects model					0.21	[0.18; 0.25]	--	38.4%
Heterogeneity: I-squared=30.3%, tau-squared=0.024, p=0.1343								
H1c								
Pittman 2005(US)	114	476	20	262	3.81	[2.31; 6.30]	0.5%	3.1%
Pittman 2005(UK)	40	166	38	338	2.51	[1.53; 4.09]	0.5%	3.1%
Cruchaga 2009	46	254	53	380	1.36	[0.89; 2.10]	0.9%	3.2%
Fixed effect model		896		980	2.31	[1.77; 3.02]	1.9%	--
Random effects model					2.33	[1.28; 4.25]	--	9.5%
Heterogeneity: I-squared=79.3%, tau-squared=0.2234, p=0.008								
Fixed effect model		15830		35392	0.94	[0.90; 0.99]	100%	--
Random effects model					0.68	[0.48; 0.97]	--	100%
Heterogeneity: I-squared=97.5%, tau-squared=0.9325, p=0.0001								

Supplementary Figure 8 Meta-analysis of the *MAPT* polymorphisms on corticobasal degeneration

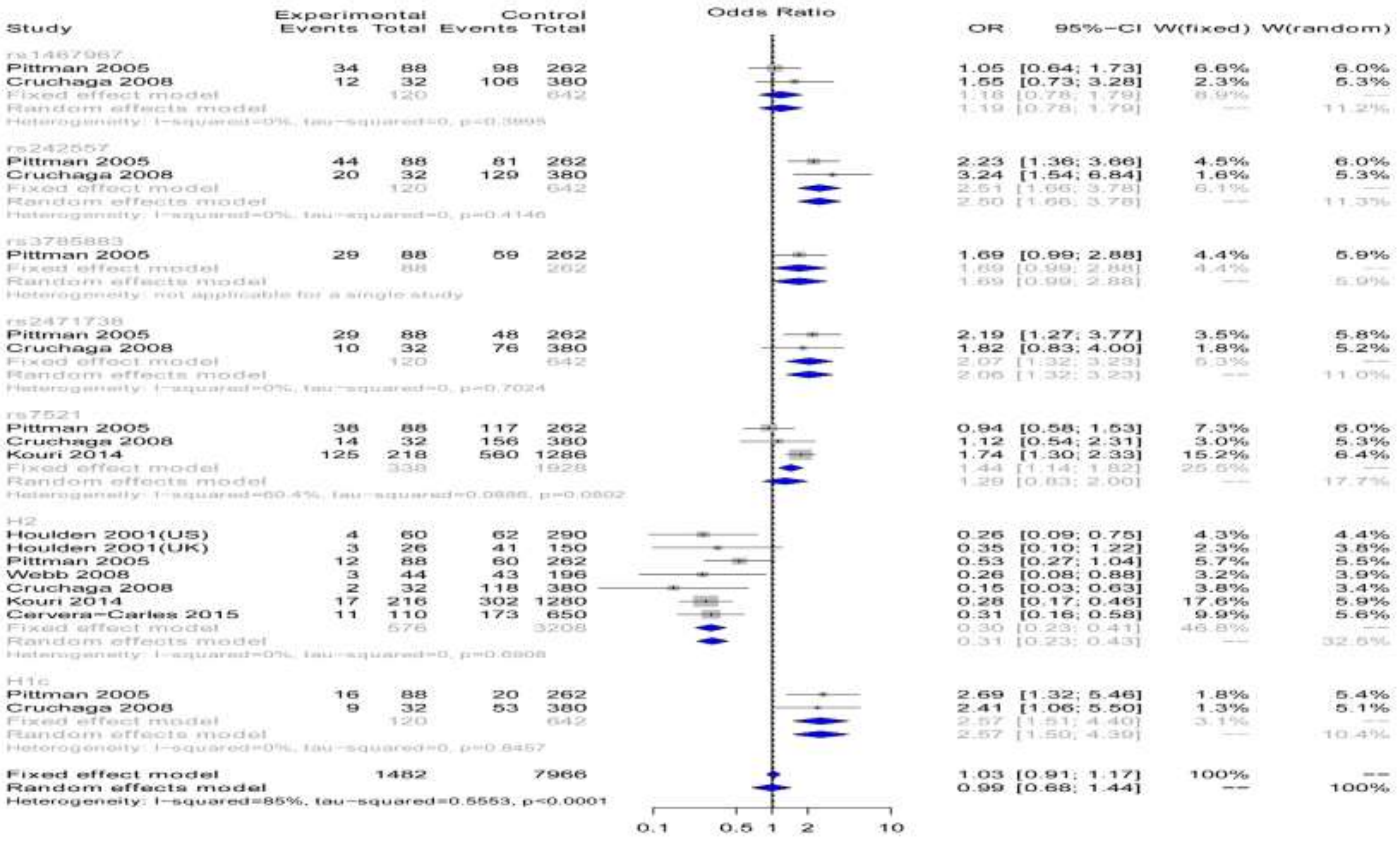

Supplementary Figure 9 Meta-analysis of the H2 haplotype on frontotemporal dementia

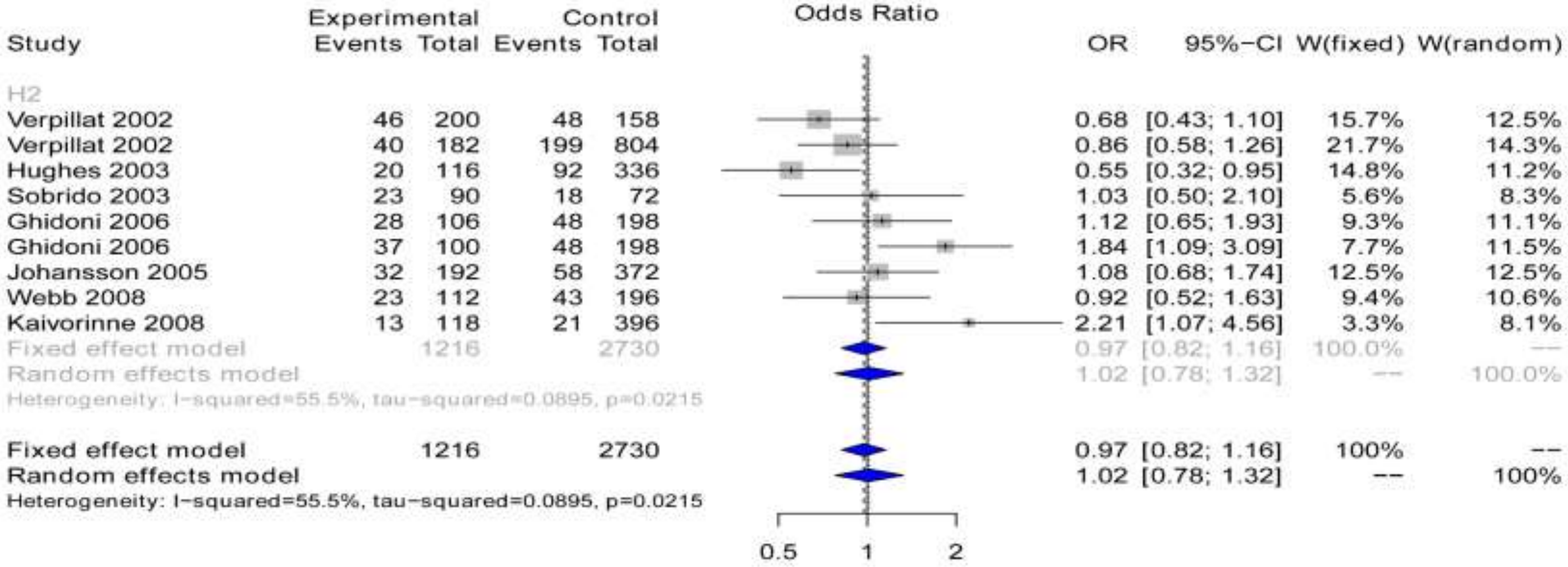

Supplementary Figure 10 Meta-analysis of the H2 haplotype on amyotrophic lateral sclerosis

Study	Experimental Events	Experimental Total	Control Events	Control Total	OR	95%-CI	W(fixed)	W(random)
H2								
Hughes 2003	49	216	92	336	0.78	[0.52; 1.16]	2.6%	2.4%
Taes 2010(Belgium)	270	684	1154	2688	0.87	[0.73; 1.03]	13.5%	13.0%
Taes 2010(Netherlands)	850	2116	892	2132	0.93	[0.83; 1.05]	25.3%	25.6%
Taes 2010(Italy)	262	532	1134	2378	1.06	[0.88; 1.28]	10.0%	10.8%
Taes 2010(Poland)	106	400	342	1074	0.77	[0.60; 1.00]	6.5%	5.8%
Taes 2010(Sweden)	260	952	260	972	1.03	[0.84; 1.26]	8.9%	9.5%
Taes 2010(US)	714	1898	758	1874	0.89	[0.78; 1.01]	22.6%	22.3%
Taes 2010(UK)	188	474	2614	6262	0.92	[0.76; 1.11]	10.6%	10.5%
Fixed effect model		7272		17716	0.92	[0.86; 0.98]	100.0%	--
Random effects model					0.92	[0.86; 0.98]	--	100.0%
Heterogeneity: I-squared=0%, tau-squared=0, p=0.452								
Fixed effect model		7272		17716	0.92	[0.86; 0.98]	100%	--
Random effects model					0.92	[0.86; 0.98]	--	100%
Heterogeneity: I-squared=0%, tau-squared=0, p=0.452								

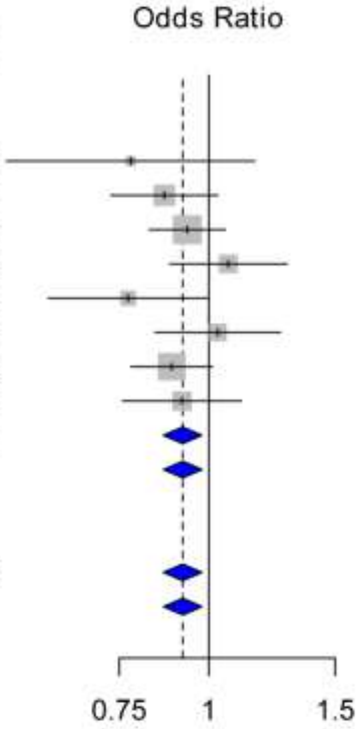

Supplement: Supplementary file 1 [file oncotarget-08-44994-s001.pdf]
